# Hyperviscous Diabetic Bone Marrow Niche Impairs BMSCs Osteogenesis via TRPV2‐Mediated Cytoskeletal‐Nuclear Mechanotransduction

**DOI:** 10.1002/advs.202509056

**Published:** 2025-12-22

**Authors:** Yao Wen, Xinhui Zheng, Jieliu Li, Minyu He, Dongqi Fan, Xingyu Zhu, Qiming Zhai, Liangjing Xin, Tao Chen

**Affiliations:** ^1^ Chongqing Key Laboratory of Oral Diseases Chongqing Municipal Key Laboratory of Oral Biomedical Engineering of Higher Education, Chongqing Municipal Health Commission Key Laboratory of Oral Biomedical Engineering Stomatological Hospital of Chongqing Medical University Chongqing 401147 P. R. China

**Keywords:** actin cytoskeleton, bone marrow mesenchymal stem cells, diabetic osteopathy, mechanotransduction, TRPV2 channel

## Abstract

The compromised regenerative capacity of diabetic bone defects remains a critical clinical challenge, with pathological alterations in the bone marrow microenvironment emerging as key contributors. While mechanical signals within the marrow niche critically regulate bone regeneration, how diabetic matrix abnormalities impair bone marrow‐derived mesenchymal stem cells (BMSCs) function remains unclear. Herein, it is revealed that diabetes induces a characteristic hyperviscous state in bone marrow extracellular matrix (ECM). Through comparative mechanobiological analyses, it is demonstrated that diabetic BMSCs exhibit amplified mechanosensitivity to ECM viscosity via transient receptor potential vanilloid 2 (TRPV2) activation. This mechanotransduction cascade triggers calcium influx, which activates CaMKII and subsequently phosphorylates cofilin, thereby shifting the G‐/F‐actin equilibrium toward perinuclear F‐actin disassembly. The cytoskeletal remodeling induces nuclear envelope deformation through regulation of Lamin A/C, driving spatial rearrangement of chromatin architecture. Mechanistically, these physical nuclear changes promote perinuclear heterochromatin accumulation and enhance H3K9me3 repressive histone modification, ultimately suppressing osteogenic transcriptional programs. Importantly, TRPV2 inhibition rescued both chromatin accessibility and osteogenic potential in diabetic BMSCs. This findings establish a novel mechano‐pathological axis where diabetic ECM hyperviscosity propagates mechanical signals from cytoskeleton to chromatin through TRPV2 activation, proposing mechanomodulation as a promising therapeutic strategy for diabetic osteopathy.

## Graphical abstract

Diabetic bone marrow exhibits pathological ECM hyperviscosity that activates TRPV2‐mediated Ca^2^⁺ influx, leading to perinuclear F‐actin disassembly, nuclear deformation, and chromatin condensation. This cytoskeletal‐nuclear decoupling suppresses osteogenic differentiation of BMSCs. By uncovering a previously unrecognized mechanical pathway linking fluid viscosity to stem cell fate, this study identifies TRPV2 as a therapeutic target to restore bone regeneration in diabetic conditions.

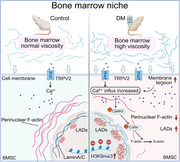

## 1 Introduction

The global diabetes mellitus (DM) epidemic, primarily driven by population aging and obesogenic lifestyles, has elevated bone fragility fractures to a major diabetic complication [1, 2]. Clinical analyses confirm a 5 fold increased fracture risk in diabetic cohorts compared to age‐matched controls, attributed to impaired osteogenic differentiation of bone marrow‐derived mesenchymal stem cells (BMSCs) and pathological marrow niche remodeling [3, 4]. As multipotent progenitors responsible for skeletal homeostasis, BMSCs exhibit lineage plasticity governed by both biochemical cues and mechanotransductive signals [5, 6]. While advances in growth factor‐mediated regulation have been well documented, the mechanobiological dysregulation underlying diabetic osteopathy remains poorly characterized—a critical knowledge gap given the essential role of physical stimuli in bone regeneration.

The bone marrow niche constitutes a dynamic 3D microenvironment where extracellular matrix (ECM) architecture, interstitial fluid dynamics, and cellular cross‐talk collectively regulate BMSCs fate decisions [7, 8]. Emerging evidence highlights fluid viscosity as a master mechanical regulator through dual mechanisms: (1) direct fluid shear stress transmission proportional to medium viscosity [9], and (2) viscosity‐dependent cytoskeletal reorganization via cell‐ECM interactions [10, 11]. Clinical observations indicate that diabetic bone marrow exhibits aberrant ECM composition, characterized by advanced glycation end‐product (AGE) accumulation and collagen crosslinking, which may elevate interstitial viscosity, strongly correlating with microangiopathy progression [12, 13]. However, whether this systemic viscosity perturbation extends to the marrow compartment to disrupt BMSCs mechanotransduction remains unverified—a paradoxical disconnect given the established viscosity sensitivity of osteoprogenitor differentiation.

Mechanistically, cells decode viscous cues through coordinated action of mechanosensors and cytoskeletal adaptors. Among these, the transient receptor potential vanilloid 2 (TRPV2), a calcium‐permeable mechanosensitive channel, has garnered attention for its tension‐dependent gating properties [14, 15]. TRPV2 dynamically regulates stem cell lineage commitment, favoring adipogenesis in compliant microenvironments by modulating Ca^2^⁺ influx and downstream transcriptional programs. However, its role in viscosity sensing and osteogenic differentiation remains poorly characterized, particularly in pathophysiological contexts such as diabetic bone marrow niches. Crucially, nuclear lamina proteins like Lamin A/C serve as physical bridges connecting cytoskeletal forces to chromatin organization [16, 17], suggesting a potential pathway through which matrix viscosity might regulate epigenetic states [18]. Nevertheless, three fundamental questions persist: 1) Does diabetic marrow develop ECM hyperviscosity? 2) How do BMSCs transduce viscous signals into chromatin‐level responses? 3) What is the functional hierarchy between ion channels and cytoskeletal elements in this mechanotransduction cascade?

Herein, for the first time, we elucidate the diabetic bone marrow as a hyperviscous mechanopathological niche and delineate a TRPV2‐centric mechanotransduction axis linking ECM viscosity to chromatin repression. Combining rheological measurements with single‐cell mechanotyping, we demonstrate that diabetic BMSCs develop hypersensitivity to matrix viscosity through TRPV2‐mediated calcium influx. This triggers perinuclear actin depletion and nuclear lamina destabilization, culminating in heterochromatin redistribution and histone‐mediated transcriptional silencing of osteogenic genes. Using a combination of TRPV2 knockdown, transcriptomic analysis, intracellular calcium chelation and ChIP‐seq assays confirms the causal role of this mechanoepigenetic pathway. Our findings position matrix viscosity as a master regulator of BMSCs differentiation and propose mechanobiological reprogramming as a therapeutic strategy for diabetic bone defects.

## 2 Result

### 2.1 Modulation of BMSCs Biological Behavior by Hyperviscous Microenvironment in Diabetic Bone Marrow

**FIGURE 1:**
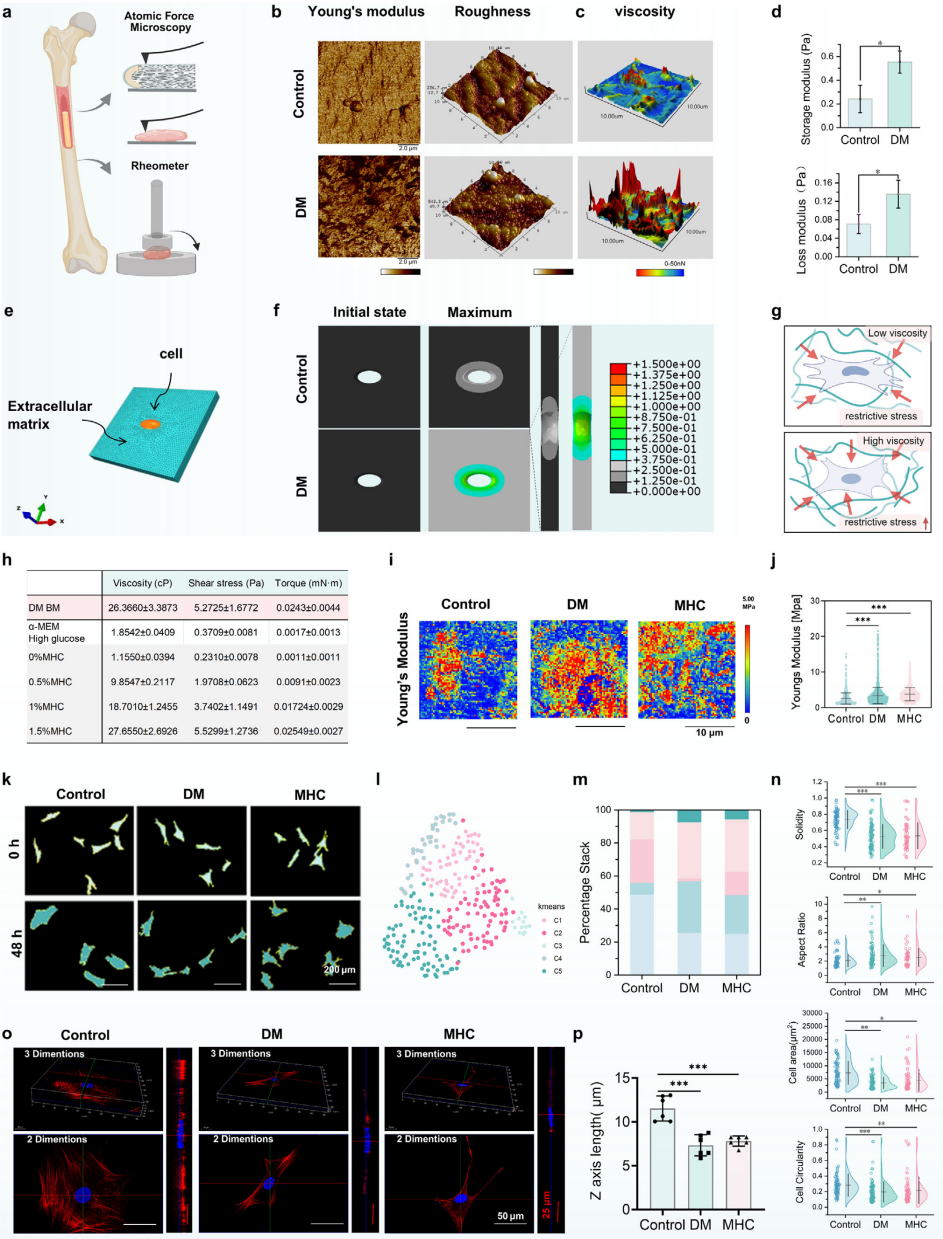
Hyperviscous niche reprograms BMSCs fate in diabetic condition. (a) Workflow integrating AFM and rotational rheometry for bone structural characterization. (b) Bone tissue stiffness quantified by Young's modulus and surface roughness. Scale bar: 20 µm (*n* = 3). (c) Retraction force‐displacement curves from AFM adhesion measurements (*n* = 3). (d) Viscoelastic properties expressed as storage (G') and loss modulus (G'') of bone marrow (*n* = 3). (e,f) ABAQUS simulation to model confining stress during cell volumetric growth with different culture conditions. (g) Schematic illustration of BMSCs under low and high viscosity microenvironment. (h) Rheometer test data table for diabetes bone marrow, α‐MEM high glucose and Methylcellulose at different concentrations, Shear rate: 200 s^−1^ (*n* = 6). (i) Single‐cell elasticity mapping using AFM measurement. Scale bar: 10 µm (*n* = 3). (j) Quantified Young's modulus stratified by treatment (*n* = 3). (k) Phase‐contrast morphology after 48 h culture. Scale bar: 200 µm (*n* = 6). (l) Unsupervised clustering categorized BMSCs under varying conditions into five distinct k‐means morphological types based on quantified shape features. (m) Bar charts illustrate the relative distribution of BMSCs across clusters (C1 to C5) under different viscosities along with representative cell morphologies. (n) Bar graphs displaying the cell perimeter length, projected cell area, circularity index, aspect ratio and cell solidity of BMSCs after culture 48 h at prescribed medium viscosity (*n* = 90). (o) 3D fluorescent imaging of F‐actin and nuclei in BMSCs. Scale bar: 50 µm for cytoskeletal proteins. 25 µm for nuclei (*n* = 6). (p) Statistical analysis of Z‐Axis length in 3D fluorescence imaging (*n* = 6). Data are presented as mean values ± standard deviation; *p < 0.05, **p < 0.01, ***p < 0.001. Two‐tailed unpaired Student's *t*‐test was used for the comparison in d), One‐way ANOVA followed by Tukey's post hoc test was used for comparisons in (j), (n), (p).

The bone marrow microenvironment serves as a critical ecological niche regulating BMSCs mechanobiology. To characterize diabetes‐induced alterations in skeletal physical properties, we systematically analyzed the biomechanical parameters of long bones using atomic force microscopy (AFM) (Figure 1a). Quantitative AFM indentation revealed a 75% elevation in Young's modulus and significant increase in surface roughness within the bone marrow cavity of diabetic rats compared to healthy controls (Figure 1b), indicating substantial matrix stiffening and structural disorganization. Complementary viscoelastic assessments through AFM‐based adhesion measurements (Figure 1c) and oscillatory rheometry demonstrated significant viscosity elevation in diabetic bone marrow. AFM retraction curves exhibited 19.72 ± 21.62 nN adhesive traction forces in diabetic specimens versus 13.28 ± 11.44 nN in controls (Figure S1a). Rheological characterization confirmed this finding, showing 129% and 86% increases in storage modulus (G′) and loss modulus (G″) of DM bone marrow tissue respectively (Figure 1d), indicating enhanced elastic energy storage and viscous dissipation characteristics in the diabetic marrow microenvironment. Building upon our characterization of diabetic marrow's elevated viscosity and matrix stiffening, we developed a 3D finite element analysis to quantify bone marrow microenvironmental stress propagation. The BMSCs system was geometrically reconstructed as a biphasic viscosity continuum, with cellular domains embedded in a rectangular tissue matrix (Figure 1e). Using bulk rheometry data and cell volume measurements, the simulation results demonstrated that DM microenvironment led to a nearly twofold increase in the overall restrictive stress on BMSCs compared to the control group (Figure 1f,g), further indicated altered mechanical energy dissipation patterns under diabetic conditions.

Conventional in vitro cell assays are generally performed using low‐viscosity culture media (approximately 1 cP). In contrast, cells in vivo reside within physiological environments exhibiting significantly higher viscosities [19]. Previous studies have reported that elevated viscosity (≥40 cP) can enhance cell motility and activate mechanotransduction pathways [20]. Moreover, under certain pathological conditions, bone marrow viscosity has been shown to reach values as high as 400 cP [21]. To establish a physiologically relevant in vitro model, we measured the viscosity of bone marrow from diabetic rats using a rheometer at 37 °C and a shear rate of 200 s^−1^. Parallel measurements were performed on culture media supplemented with varying concentrations of methylcellulose (MHC) and high‐glucose media. The viscosity of diabetic rat bone marrow was determined to be ∼ 26.37 cP. In comparison, the viscosity of MHC‐containing media increased in a concentration‐dependent manner, with the 1.5% MHC formulation most closely matching the bone marrow viscosity measured in diabetic rats. By contrast, the high‐glucose medium exhibited viscosity comparable to that of standard medium, indicating no significant alteration (Figure 1h). Based on these rheological analyses, 1.5% MHC was selected to mimic the high‐viscosity diabetic microenvironment in subsequent experiments. This solution provides mechanical stimulation without impeding oxygen and nutrient exchange or causing excessive stress (Figure S1b). 3D finite element analysis was employed to dissociate the mechanical effects of hyperviscosity from the biochemical effects of high glucose. Our simulations revealed that the confining stress on cells in the high‐glucose group was comparable to the control. In contrast, the hyperviscous medium (1.5% MHC) induced a near two‐fold increase in overall confining stress (Figure S2a). These results demonstrate that the altered cellular stress is not a consequence of biochemical alterations but is primarily attributed to the elevated viscosity. Osteogenic function validation showed that 1.5% MHC replicated the diabetic microenvironment, reducing osteogenic differentiation and downregulating stem cell‐related genes (Figure S2b,c). Considering bone marrow viscosity, cell viability, and osteogenic efficacy, 1.5% MHC was chosen for subsequent experiments. Notably, MHC groups showed an increased SA‐β‐Gal^+^ cell proportions (Figure S2d) yet paradoxically enhanced migration capacity and transwell penetration, suggesting viscosity‐triggered mesenchymal transition (Figure S2e–g). Subcellular ultrastructural quantification demonstrated mitochondrial pathology: cristae density decreased with matrix swelling (Figure S2h), indicative of metabolic reprogramming. These data collectively demonstrate that diabetic‐level viscosity induces BMSCs fate regression through coordinated suppression of osteogenic commitment, stemness maintenance, and mitochondrial homeostasis, while promoting senescence‐associated phenotypes and aberrant motility.

AFM nanoindentation analysis revealed that BMSCs subjected to elevated extracellular confinement stress exhibited enhanced intrinsic resistance to mechanical loading, with Young's modulus increasing by 46% (MHC: 3.751 ± 1.842 nN) and 30% (DM: 3.35 ± 2.292 nN) compared to controls (2.565 ± 1.570 nN; Figure 1i,j). This stiffness elevation exhibited strong correlation with microenvironmental viscosity, mechanistically linking extracellular viscosity loading to cellular mechanotransduction responses. Subsequently, the morphological evolution of BMSCs across three experimental groups was systematically evaluated at 0‐ and 48 h post‐culture initiation. Quantitative analysis revealed a significant volumetric expansion in normal cultures compared to their high‐viscosity counterparts (Figure 1k). To comprehensively characterize viscosity‐mediated morphological adaptations, we implemented a multi‐parametric assessment framework following 48 h culture periods. The morphology of the cells after 48 h in vitro culture is shown in Figure 1k, fixed BMSCs were subjected to DIO membrane staining, enabling precise boundary demarcation for subsequent quantification of four critical morphological parameters: 1) cell solidity, 2) aspect ratio (describing deviation from a perfect circle), 3) projected cell area and 4) circularity index (Figure 1n). Comparative analysis demonstrated remarkable similarity between DM and MHC groups, with both exhibiting significant divergence from control specimens across four morphological descriptors. K‐means clustering based on cell morphological scores identified five morphological clusters with unique cellular features, Uniform Manifold Approximation and Projection (UMAP) dimensionality reduction effectively revealed viscosity‐dependent morphological signatures within the BMSCs populations (Figure 1l). Cluster‐based quantification of five morphological domains revealed distinct patterning: high‐viscosity cultures exhibited phenotypic convergence with diabetic‐derived cells, while normal conditions generated unique morphological profiles distinct from both DM and MHC groups (Figure 1m). Spatial distribution analysis showed normal BMSCs predominantly occupying clusters C2/C3, contrasting with the C1/C3/C4 predominance observed in DM and MHC specimens. To analyze 3D cellular morphology, we performed co‐staining of cytoskeletal proteins and nuclei. Quantitative Immunofluorescence analysis revealed that both the overall and nuclear height of cells in the control group were significantly greater than those in the other two conditions (Figure 1o), a conclusion further supported by statistical analysis (Figure 1p). The results establish pathological elevation of bone marrow viscosity as a critical biomechanical regulator in diabetes, driving BMSCs characteristic morphological patterning and dysfunction.

### 2.2 Hyperviscous Microenvironment Leads to Actin Rearrangement and Abnormal Lamin A/C Morphology of BMSCs under Diabetic Conditions

**FIGURE 2:**
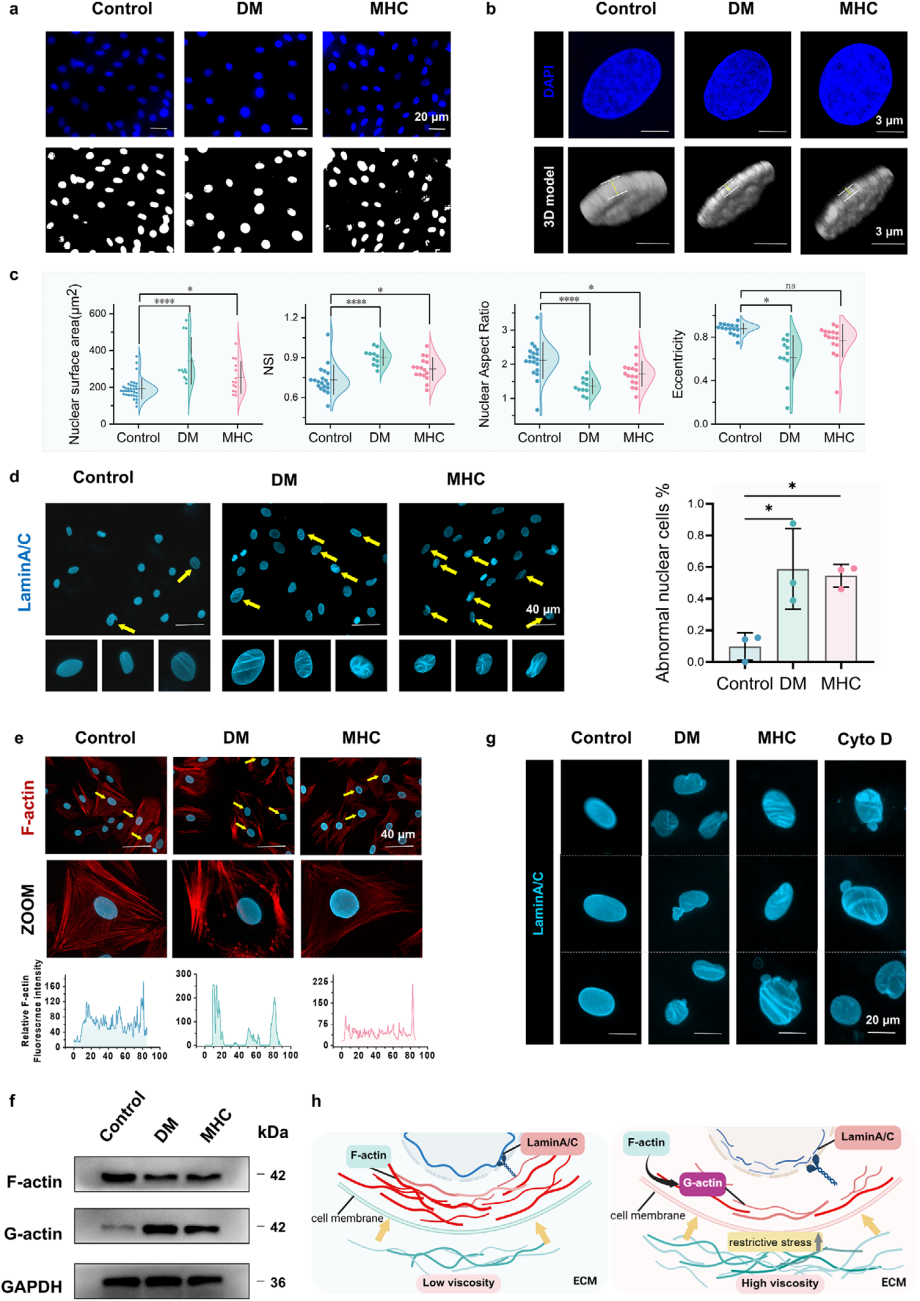
Hyperviscosity‐induced cytoskeletal remodeling drives nuclear envelope pathology in BMSCs. (a) Nuclear architecture analysis: DAPI staining (upper) and ImageJ‐based nuclear segmentation (lower) of nucleus. Scale bar: 20 µm (*n* = 11–19). (b) 3D nuclear reconstruction from z‐stack confocal images. Scale bar: 3 µm (*n* = 7). (c) Quantitative nuclear morphometrics: nuclear surface area, nuclear aspect ratio, nuclear shape index (NSI) and eccentricity (*n* = 11–19). (d) Lamin A/C immunofluorescence with abnormal nuclear envelope quantification. Scale bar: 40µm (*n* = 3). (e) Immunofluorescence image of F‐actin and nuclear. Normalized fluorescence intensity profiles of F‐actin across the nucleus (bottom). Scale bar: 40 µm (*n* = 3). (f) Expression of F‐actin and G‐actin quantified by western blotting. (g) Immunofluorescence staining images of BMSCs Lamin A/C under different treatments. Scale bar: 20 µm (*n* = 3). (h) Schematic illustration of the relationship between F‐actin and Lamin A/C in different viscous condition. All data are presented as mean values ± standard deviation; *p < 0.05, **p < 0.01, ***p < 0.001, ****p < 0.0001. One‐way ANOVA followed by Tukey's post hoc test was used for comparisons in (c), (d).

Mechanosensitive signaling networks mediate cellular interpretation of biomechanical microenvironmental changes by transducing physical stimuli into biochemical signals [22, 23]. Accumulating evidence confirms that the nucleus serves as a critical mechanotransduction hub, directly sensing mechanical stress and strain to regulate cellular functions [24]. Quantitative analysis of nuclear morphological parameters revealed distinct deformation patterns in DM and MHC groups compared to controls. Specifically, both experimental groups demonstrated statistically significant increases in nuclear area and nuclear shape index (NSI), accompanied by decreased nuclear aspect ratio, while nuclear eccentricity remained no significant difference across all groups (Figure 2a,c). 3D reconstruction further demonstrated vertical nuclear expansion in DM and MHC groups (Figure 2b), statistical analysis of nuclear height also revealed that the Control group exhibited higher nuclear height compared to the other two groups (Figure S3a), collectively suggesting that nuclear mechanoadaptation involves multidimensional morphological adjustments in response to extracellular viscosity variations. This mechanical adaptation appears associated with nuclear envelope (NE) integrity [25, 26]. Lamin A/C, a key nuclear envelope protein maintaining nuclear stability, has been previously shown to regulate BMSCs osteogenic differentiation potential through expression level modulation [18, 27]. Our immunofluorescence analysis revealed characteristic NE abnormalities in experimental groups, including increased incidence of envelope wrinkling, indentation, and lobulation. Quantitative assessment confirmed significantly higher proportions of abnormal nuclear envelope structures in DM and MHC groups compared to controls (Figure 2d). The structural integrity of nuclear morphology is maintained by the apical perinuclear actin cap‐a dome‐shaped F‐actin network enveloping the nuclear apex that coordinates NE expansion while preventing pathological deformation [28, 29]. Immunofluorescence analysis revealed distinct F‐actin spatial reorganization patterns across experimental groups: control cells displayed uniform F‐actin distribution with dense perinuclear enrichment, whereas DM and MHC groups exhibited polarized F‐actin redistribution characterized by front‐edge accumulation and perinuclear depletion (Figure 2e). To further assess cytoskeletal remodeling, we quantified F‐actin and G‐actin levels by western blot. The analysis revealed a marked depolymerization of F‐actin in the DM and MHC groups, as evidenced by decreased F‐actin and a concomitant increase in monomeric G‐actin, indicating a shift in the equilibrium toward the globular form (Figure 2f).

This mechanoadaptive response aligns with established cytoskeletal stress‐shielding mechanisms, where cortical F‐actin assembly counteracts elevated extracellular confinement stresses in 3D microenvironments. To figure out causality between perinuclear actin dynamics and nuclear envelope integrity, we employed Cytochalasin D (Cyto D), an F‐actin‐depolymerizing agent. Quantitative analysis of F‐actin architecture revealed distinct spatial reorganization patterns: control cells maintained uniform F‐actin distribution with significant perinuclear enrichment, whereas Cyto D‐treated controls exhibited cortical F‐actin accumulation alongside marked perinuclear depletion, mirroring the pathological redistribution observed in DM and MHC groups (Figure S3b). Pharmacological disruption of the actin cap in control group induced characteristic NE abnormalities, transforming Lamin A/C morphology from smooth ovoid profiles to concave, lobulated configurations (Figure 2g). These findings mechanistically demonstrate that viscosity‐dependent actin cap disassembly compromises NE structural fidelity through Lamin A/C disorganization (Figure 2h). Parallel evaluation of YAP subcellular localization demonstrated a cytoplasmic shift across DM and MHC groups, with nuclear/cytoplasmic ratios decreasing compared to controls (Figure S3c), The addition of Cyto D in the control group reduced the nucleoplasmic localization (Figure S3d). This mechanistically linked the actin‐mediated nuclear stabilization to YAP nucleocytoplasmic shuttling efficiency.

### 2.3 Calcium Elevation Drives Cytoskeletal Remodeling in Diabetic and Hyperviscous Microenvironment

**FIGURE 3:**
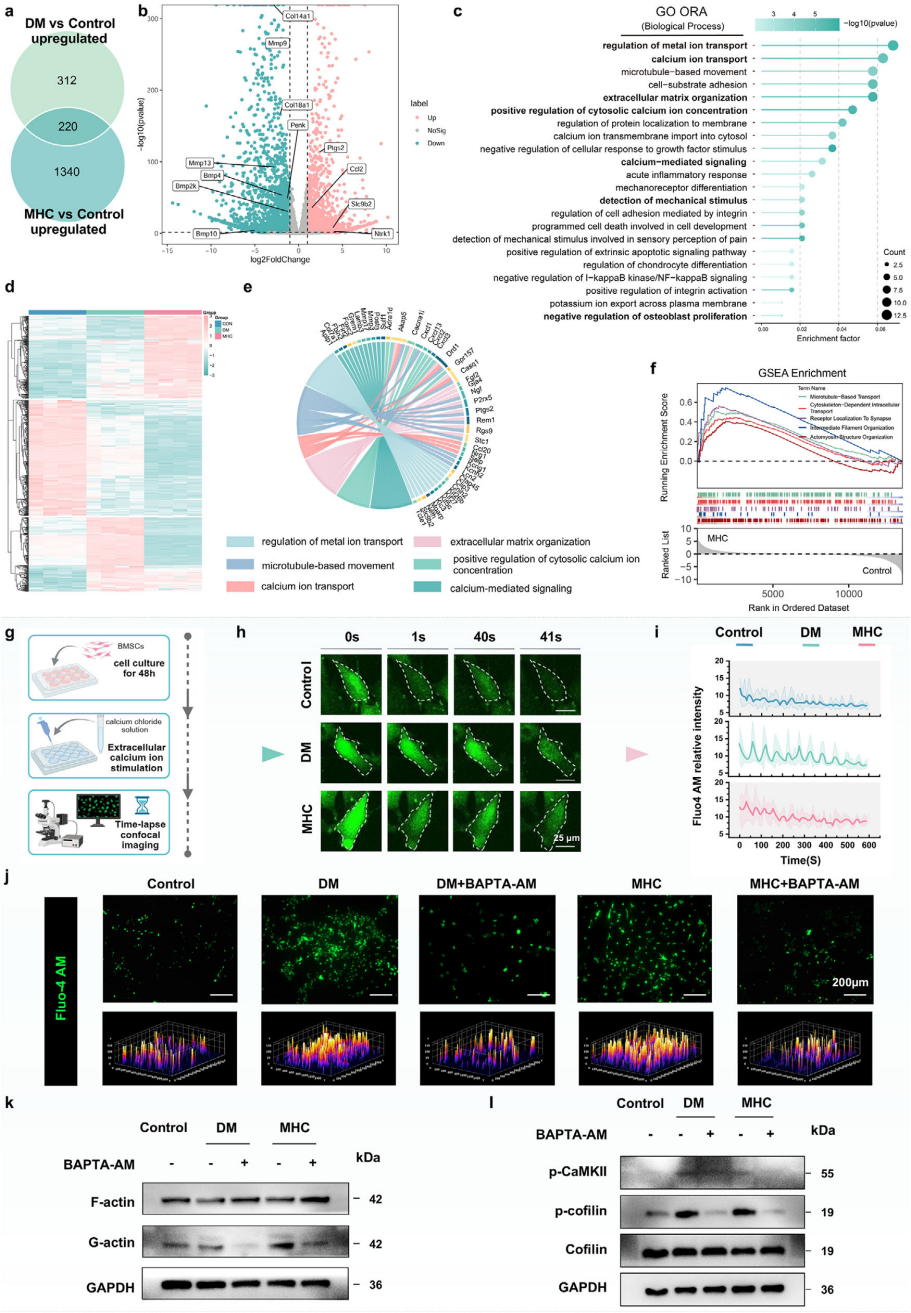
Calcium dysregulation and cytoskeletal changes in diabetes under hyperviscous microenvironment. (a) Intersectional analysis via Venn diagram (*n* = 4). (b) Volcano plot of DEGs between the MHC and Control groups (*n* = 4). (c) GO enrichment of DEGs (*n* = 4). (d) Hierarchical clustering analysis of DEGs. Genes were clustered into three clusters via K‐means clustering (*n* = 4). (e) Chord diagram of DEGs involved in calcium ion transport and other related biological processes (*n* = 4). (f) The GSEA of the MHC group in comparison to the Control group (*n* = 4). (g–i) Quantitative analysis of calcium signal oscillations. Scale bar: 25 µm (*n* = 4). (j) Representative images of cytosolic calcium. Scale bar: 200 µm (*n* = 4). (k) Expression of F‐actin and G‐actin quantified by western blotting. BMSCs were treated with BAPTA‐AM (50 µM) to prevent calcium overload. (l) Expression of p‐CaMKII and p‐cofilin quantified by western blotting. All data are presented as mean values ± standard deviation.

To delineate conserved pathomechanical signatures under hyperviscosity, we performed RNA‐seq profiling of BMSCs across experimental cohorts. 3D principal component analysis (PCA) demonstrated pronounced transcriptomic heterogeneity across groups, with the first two principal components resolving discrete clustering between Control and DM/MHC groups. Tight intra‐group clustering and significant inter‐group separation confirmed distinct expression signatures (Figure S4a). Intersectional analysis via Venn diagram revealed 220 co‐upregulated genes in DM/MHC (Figure 3a). Volcano plot analysis identified differentially expressed genes (DEGs) in MHC versus Control, comprising 1645 upregulated and 2145 downregulated targets. Notably, osteogenic suppressors and matrix remodeling enzymes showed conserved dysregulation patterns in both DM and MHC groups (Figure 3b). Next, GO enrichment analysis of shared DEGs highlighted mechanopathologically relevant pathways, including regulation of metal ion transport, calcium ion transport, extracellular matrix organization and negative regulation of osteoblast proliferation (Figure 3c). Hierarchical clustering categorized these DEGs into three distinct clusters (Figure 3d), revealing reversed gene expression patterns in MHC group compared to the Control group. Chord diagram visualization quantified pathway interconnectivity, showing calcium signaling and ECM remodeling as central hubs interacting with DEGs (Figure 3e). GSEA confirmed cytoskeletal pathway activation in MHC group (Figure 3f), establishing hyperviscosity‐induced cytoskeletal reprogramming as a hallmark of diabetic osteopathy.

To assess calcium channel activity dynamics under DM and MHC conditions, we employed quantitative live‐cell imaging with Fluo‐4 AM. Cells subjected to DM/MHC microenvironments exhibited sustained [Ca^2^⁺]_i_ overload, demonstrating higher mean fluorescence intensity compared to controls (Figure S4b). Flow cytometric quantification corroborated this calcium dysregulation, revealing an increase in Fluo‐4⁺ cell population (Figure S4c). Furthermore, quantitative analysis of calcium signal oscillations revealed that individual cells cultured under DM and MHC conditions exhibited higher calcium oscillation amplitudes compared to the Control group (Figure 3g–i), suggesting viscosity‐dependent potentiation of calcium signaling through sustained channel activation. To investigate the functional significance of elevated calcium signaling in DM and MHC groups, we applied the calcium chelator BAPTA‐AM. Immunofluorescence confirmed markedly reduced calcium‐dependent fluorescence intensity after treatment (Figure 3j). We further examined whether F‐actin depolymerization was linked to intracellular calcium levels. Western blot showed that BAPTA‐AM restored F‐actin expression (Figure 3k), supporting calcium‐dependent cytoskeletal remodeling. Given that cofilin is inactivated via phosphorylation at Ser‐3 through Ca^2^⁺/CaMKII signaling [30, 31], we assessed this pathway. Enhanced phosphorylation of CaMKII and cofilin in DM and MHC groups was reversed by BAPTA‐AM (Figure 3l), confirming calcium dependence.

Collectively, these findings establish a mechanistic cascade whereby a high‐viscosity microenvironment elevates intracellular Ca^2^⁺ levels, leading to CaMKII activation, subsequent cofilin phosphorylation, and ultimately F‐actin depolymerization.

### 2.4 TRPV2‐Dependent Calcium Signaling Regulates Cytoskeleton and Nuclear Envelope in HighViscosity Conditions

**FIGURE 4:**
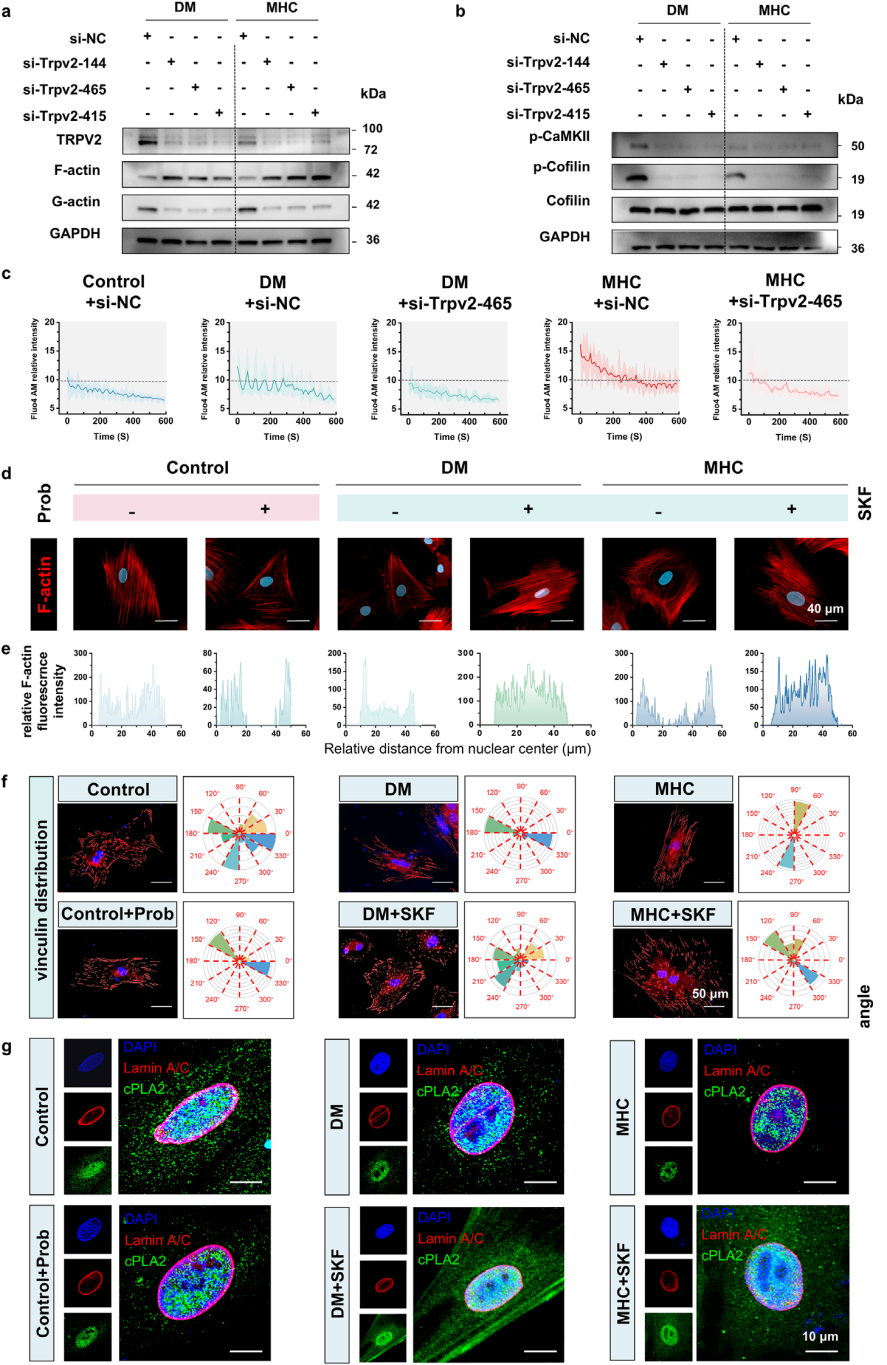
TRPV2‐mediated mechanosensing coordinates actomyosin‐Lamin A/C remodeling under hyperviscosity. (a) Expression of F‐actin and G‐actin quantified by western blotting, TRPV2 was knocked down in BMSCs using siRNA technology. (b) Expression of CaMKII and cofilin quantified by western blotting. (c) Quantitative analysis of calcium oscillations after si‐TRPV2 treatment (*n* = 4). (d) Immunofluorescence images of F‐actin and nuclear. Scale bar: 40 µm (*n* = 3). (e) Normalized F‐actin intensity profiles across nuclear equator. (f) Immunofluorescence image of vinculin focal adhesion angular distribution. Scale bar: 50 µm. (g) Dual‐channel confocal imaging of cPLA2 (Green) and Lamin A/C (Red). Scale bar: 10 µm (*n* = 4).

As a mechanosensitive member of the TRP channel superfamily, the homotetrameric N‐glycosylated TRPV2 transduces extracellular mechanical stress into intracellular calcium signals through its membrane‐spanning pore architecture [32]. Transcriptional profiling and immunofluorescence analysis confirmed consistent TRPV2 upregulation under DM and MHC conditions (Figure S5a,b). To functionally define TRPV2's role in viscosity‐sensing, we employed both pharmacological and genetic approaches. Pharmacological inhibition of TRPV2 with SKF‐96365 (SKF) significantly attenuated F‐actin depolymerization, a finding further corroborated by TRPV2 knockdown using siRNA (si‐TRPV2) in BMSCs under hyperviscosity (Figure 4a; Figure S5c,d). This genetic suppression of TRPV2 not only diminished F‐actin depolymerization but also led to a marked reduction in phosphorylation of downstream effectors CaMKII and cofilin (Figure 4b). Consistently, TRPV2 knockdown substantially suppressed both the frequency and amplitude of intracellular Ca^2^⁺ transients (Figure 4c), underscoring its critical role in mediating mechanosensitive calcium signaling and subsequent actin cytoskeletal remodeling. Immunofluorescence analysis of perinuclear actin architecture revealed TRPV2‐dependent cytoskeletal reorganization. Pharmacological activation with Prob in control groups induced reduction in apical F‐actin density with peripheral redistribution, while SKF treatment restored physiological F‐actin distribution in DM/MHC groups (Figure 4d,e). Calcium flux analysis demonstrated that Prob treatment in control groups induced higher [Ca^2^⁺]_i_ elevation, while SKF administration in DM/MHC groups normalized calcium overload of control levels (Figure S5e). Transwell migration assays further confirmed TRPV2's mechanosensitive role—SKF suppressed hyperviscosity‐driven migration, while Prob amplified migratory capacity versus untreated controls (Figure S5e). Quantitative morphometrics confirmed TRPV2 functions as a mechanostructural coordinator, mediating viscosity‐induced nuclear envelope remodeling through actin‐nucleus force transduction. As key mechanotransductive interfaces, focal adhesions (FAs) facilitate the coupling of extracellular biomechanical cues with the intracellular actin cytoskeleton through dynamic reorganization [33, 34]. Quantitative analysis of vinculin spatial patterning revealed distinct organizational modes across conditions. The Control+Prob, DM, and MHC groups exhibited constrained angular dispersion of vinculin. Pharmacological intervention with SKF promoted a more distributed pattern (Figure 4f). This variation in vinculin organization was associated with the state of the actin cytoskeleton; a more polarized vinculin alignment correlated with enhanced stress fiber polarization and improved directional migration capacity (Figure S2e–g). These observations suggest that the spatial patterning of vinculin, while not a comprehensive readout of FA maturity, may serve as a correlative marker for the cytoskeletal remodeling that underlies changes in cell motility.

Cytosolic phospholipase A2 (cPLA2), a calcium‐responsive mediator of nuclear‐cytoplasmic communication, undergoes Ca^2^⁺‐dependent translocation to the nuclear envelope during mechanostress‐induced cellular/nuclear expansion [35, 36]. Immunofluorescence quantification demonstrated that Control and SKF‐normalized groups (DM+SKF, MHC+SKF) exhibited diffuse cytoplasmic cPLA2, whereas hyperviscosity‐challenged groups (Control+Prob, DM, MHC) showed pronounced nuclear membrane accumulation (Figure 4g). This translocation strongly correlated with [Ca^2^⁺]_i_ elevation, mechanistically linking calcium overload to nuclear envelope tension modulation. Concomitant Lamin A/C analysis revealed inverse spatial coordination‐nuclear envelope‐associated cPLA2 intensity increased while Lamin A/C signal decreased in pathological groups, indicating calcium‐mediated nuclear softening (Figure S5f).

Collectively, our findings establish TRPV2 as a mechanotransduction nexus wherein hyperviscosity‐triggered calcium influx orchestrates cytoskeletal‐nuclear uncoupling through perinuclear actin disassembly, inducing nuclear envelope remodeling and Lamin A/C disorganization (Figure S5g).

### 2.5 Hyperviscosity‐Driven TRPV2 Activation Reprograms Chromatin Architecture in Diabetic Bone Regeneration

**FIGURE 5:**
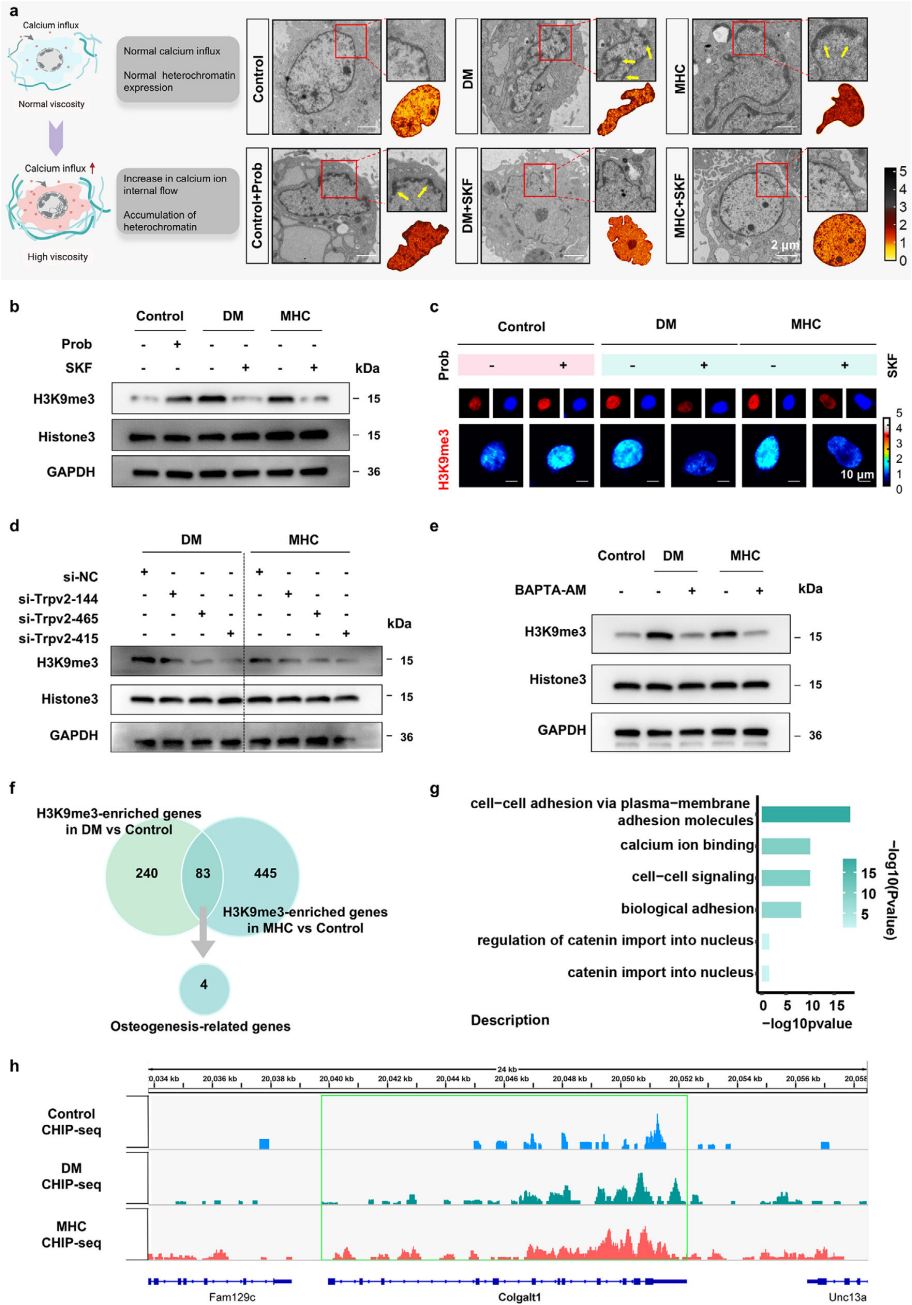
Hyperviscous microenvironment drives chromatin remodeling through calcium‐mediated mechanotransduction to impair osteoregeneration. (a) Chromatin Transmission Electron Microscopy of Lamin‐associated domains (LADs), with chromatin density heatmaps generated by ImageJ. Scale bar: 2 µm (*n* = 3). (b) Expression of H3K9me3 quantified by western blotting. (c) H3K9me3 immunostaining with DAPI counterstain. Scale bar: 10 µm (*n* = 3). (d) Expression of H3K9me3 quantified by western blotting in BMSCs after si‐TRPV2 treatment. (e) After treatment with BAPTA‐AM, expression of H3K9me3 quantified by western blotting. (f) Intersectional analysis via Venn diagram (*n* = 2). (g) GO enrichment analysis of DEGs in the MHC group (*n* = 2). (h) Representative Integrative Genomics Viewer (IGV) tracks demonstrating increased H3K9me3 binding enrichment within heterochromatic regions of the Colgalt1 gene in the DM and MHC groups compared to the control (*n* = 2).

Genomic loci positioned at the nuclear periphery typically exhibit transcriptional quiescence, with lamina‐associated domains (LADs) constituting heterochromatic regions anchored to the nuclear lamina. These domains undergo dynamic reorganization in response to perturbations in Lamin A/C integrity and nuclear morphology, resulting in altered peripheral chromatin compaction [16, 37]. Using ChromTEM analysis, we observed a marked increase in perinuclear chromatin density in BMSCs from DM and MHC groups compared to Control group (Figure 5a). Notably, TRPV2 activation (via Prob) in Control group or pharmacological inhibition (via SKF) in DM/MHC groups reversed chromatin condensation, implicating viscosity‐driven TRPV2‐dependent calcium influx as a mechanistic regulator of nuclear skeleton reorganization. Given the established correlation between LADs and transcriptional silencing through enrichment of repressive histone marks, we interrogated chromatin states using histone modification markers [38].

Western blot analysis showed increased levels of the repressive histone mark H3K9me3 in DM, MHC, and Prob groups, which was reversed by SKF treatment (Figure 5b). A similar pattern was observed for H3K27me3 (Figure S6a), whereas the activating mark H3K4me3 showed an opposite trend (Figure S6b). Immunofluorescence analysis further revealed heterochromatin consolidation in these groups, characterized by enhanced H3K9me3 and reduced H3K14ac signals, with SKF restoring a euchromatin state similar to controls (Figure 5c; Figure S6c). Genetic knockdown of TRPV2 using si‐TRPV2 significantly suppressed H3K9me3 upregulation in DM and MHC groups (Figure 5d), and intracellular calcium chelation with BAPTA‐AM similarly inhibited H3K9me3 elevation (Figure 5e). Together, these results demonstrate that the hyperviscous microenvironment drives chromatin remodeling through a TRPV2‐mediated calcium signaling axis.

To identify the specific genes targeted by H3K9me3 methylation that may contribute to the reduced osteogenic potential of BMSCs, we performed chromatin immunoprecipitation sequencing (ChIP‐seq) for H3K9me3. An intersectional analysis of H3K9me3‐enriched genes in the DM and MHC groups versus control identified 88 common targets, four of which are closely associated with osteogenic differentiation (Figure 5f). GO pathway analysis revealed significant enrichment in pathways including “intercellular adhesion” and “calcium ion binding” in the MHC group (Figure 5g), while the DM group showed enrichment in “alternative ossification”, “intercellular adhesion”, and “skeletal system development” (Figure S7a). Among the four osteogenesis‐related genes, Colgalt1 (collagen β‐1,4‐galactosyltransferase 1) participates in collagen modification and bone matrix formation (Figure 5h). Its reduced expression may compromise collagen stability, increase tissue fragility, and perturb cellular signaling via alterations in the extracellular matrix, thereby linking it to impaired osteogenesis. Our ChIP‐seq results confirmed elevated H3K9me3 binding in the heterochromatic regions of Colgalt1, as well as in Gabrb1, Plec and Spon1 in both DM and MHC groups. While Spon1 and Gabrb1 are directly associated with osteogenesis, Plec encodes plectin, a key protein that facilitates the critical linkage between the cytoskeleton and the nuclear skeleton via its interaction with nesprin‐3 (Figure S7b–d).

**FIGURE 6:**
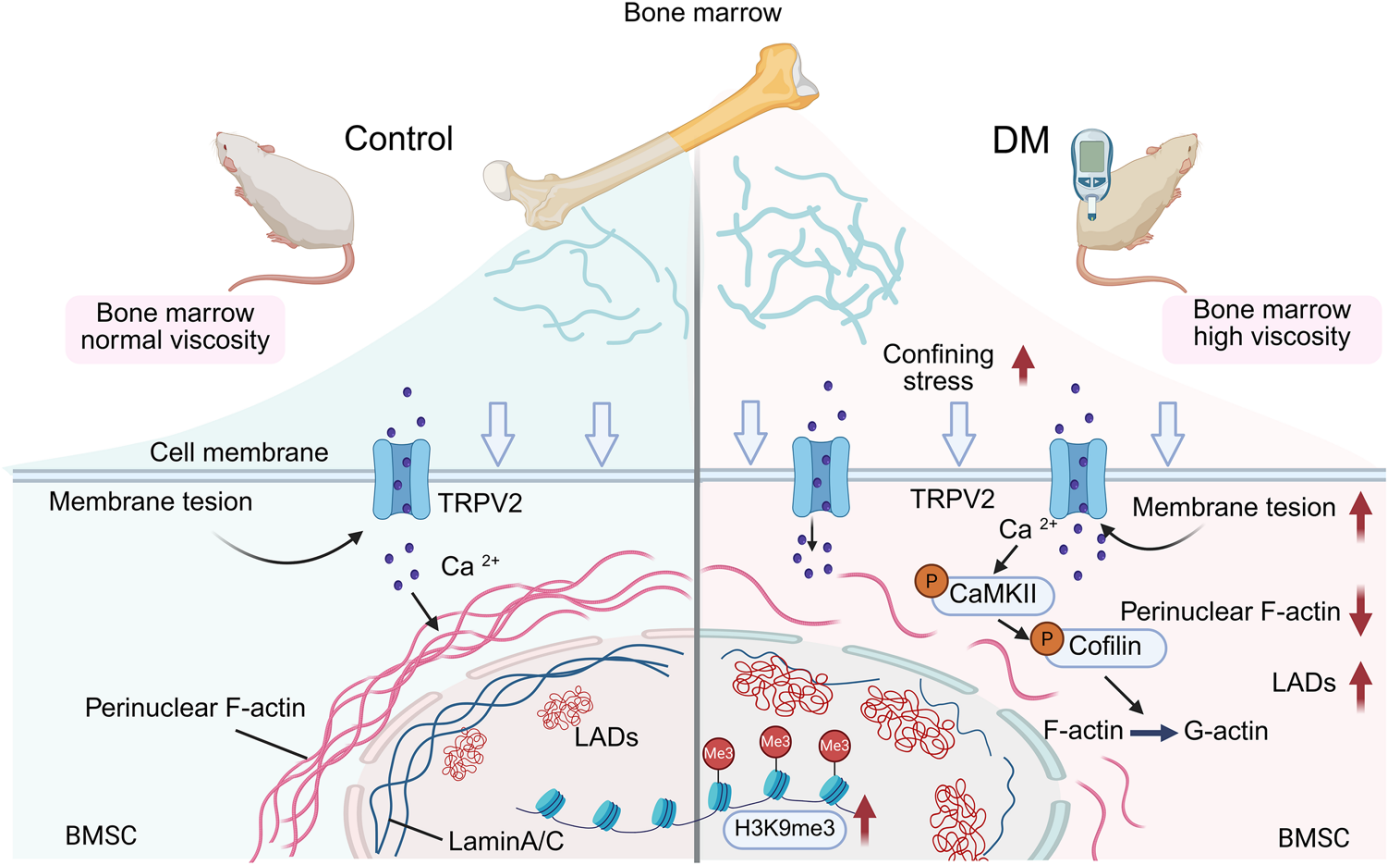
Schematic illustration of mechanotransduction in BMSCs within a diabetic high viscosity marrow microenvironment. In the high viscosity niche, elevated confining stress imposes loading on the plasma membrane, gating the mechanosensitive Ca^2^⁺ channel TRPV2. The resultant Ca^2^⁺ influx triggers CaMKII phosphorylation, which in turn phosphorylates cofilin, shifting the G‐/F‐actin equilibrium toward perinuclear F‐actin disassembly. This cytoskeletal remodeling deforms Lamin A/C and repositions lamina‐associated domains (LADs), ultimately expanding H3K9me3‐marked heterochromatin and driving transcriptional repression of osteogenic genes.

As shown in Figure S8, PCR analysis and ALP staining revealed significant downregulation of osteogenic genes in both DM and MHC groups compared to controls, consistent with hypermethylation‐associated transcriptional suppression. Osteogenic capacity was restored upon pharmacological inhibition or genetic knockdown of TRPV2 (Figure S8a,b). Furthermore, we administered Prob to wild‐type rats and SKF to diabetic rats in a calvarial defect model to validate TRPV2's pathophysiological role (Figure S8c). Strikingly, Prob exacerbated cranial defect areas in WT animals, while SKF significantly enhanced bone regeneration in diabetic cohorts (Figure S8d). The above evidence corroborates our hypothesis that hyperviscosity‐induced TRPV2 activation compromises BMSCs' reparative capacity, proposing TRPV2 modulation as a therapeutic strategy to ameliorate diabetes‐impaired osteogenesis (Figure 6).

## 3 Discussion

While extensive research has focused on the role of matrix stiffness and surface topography in regulating BMSCs osteogenesis, the mechanobiological interplay between extracellular fluid dynamics and stem cell fate determination remains poorly characterized. Emerging evidence implicates fluid viscosity as a key modulator of cellular mechanosensing [39, 40], yet its pathological implications in diabetic bone marrow niches are unexplored. Notably, clinical evidence highlights the bone marrow as a critical yet understudied site of end‐organ damage in diabetes [41, 42], where pathological alterations in interstitial fluid properties, including hyperviscosity due to AGEs accumulation, may profoundly impair BMSCs functionality.

For the first time, our study reveals that diabetic bone marrow exhibits a hyperviscous microenvironment, as evidenced by AFM‐based quantification of elevated viscosity in marrow, corroborating the abnormal hemorheological properties in diabetes. To model this pathophysiological condition in vitro, we engineered methylcellulose‐supplemented media to recapitulate diabetic level viscosity, demonstrating that hyperviscosity impairs BMSCs osteogenic differentiation through amplified mechanical stress transmission. Mechanistically, hyperviscosity triggers TRPV2‐dependent Ca^2^⁺ influx, inducing nuclear envelope remodeling and Lamin A/C disorganization. This force transmission drives nuclear deformation and subsequent H3K9me3‐mediated chromatin condensation, ultimately suppressing osteogenic gene transcription. These findings position matrix viscosity as a pivotal biomechanical regulator distinct from conventional stiffness‐driven mechanotransduction pathways.

The emerging research of mechanobiology highlights the pivotal role of fluid dynamics in modulating cellular physiology through mechanosensitive ion channels [43, 44]. Our findings position TRPV2, a tension‐gated calcium channel, as a central mechanotransducer in diabetes‐associated bone marrow hyperviscosity. By driving TRPV2 overexpression and consequent calcium overload, this pathological microenvironment initiates a self‐amplifying cascade of cytoskeletal remodeling. Key features include phosphorylation of CaMKII and cofilin, perinuclear F‐actin disassembly into G‐actin, a pattern which recapitulates the stress‐shielding adaptation seen in confined 3D matrices [45]. The biomechanical perturbations propagate bidirectionally: while extracellular viscosity modulates TRPV2 activity, the channel reciprocally regulates nuclear mechanopathology. Our results revealed that TRPV2 activation induces nuclear envelope invagination and Lamin A/C disorganization. Notably, the disruption of nuclear‐cytoskeletal connectivity suggests a potential role for linker of nucleoskeleton and cytoskeleton (LINC) complex proteins, such as nesprins and SUN proteins, in mediating force transmission under hyperviscous stress. Future studies should investigate whether hyperviscosity alters the expression or localization of these critical auxiliary proteins, which may further compromise nuclear mechanostability and gene regulation. This mechanical uncoupling disrupts nuclear‐cytoskeletal connectivity, the protective effect of perinuclear actin depletion on nuclei is decreased. Notably, these findings resonate with recent work showing voltage‐gated calcium channel‐actin interactions regulate calcium channel clearance via force‐dependent cytoskeletal remodeling, suggesting conserved mechanoregulatory principles across ion channel families [46].

Chromatin spatial reorganization emerges as the terminal effector of this mechano‐epigenetic axis, with ChromTEM mapping demonstrating perinuclear heterochromatin accumulation alongside elevated H3K9me3 repressive marks. This epigenetic reprogramming correlates with transcriptional repression of osteogenic‐related genes, recapitulating the osteogenic suppression observed in diabetic osteopathy [47, 48]. Chromatin immunoprecipitation sequencing (ChIP‐seq) targeting H3K9me3 methylation further confirmed elevated H3K9me3 binding levels in heterochromatic regions of osteogenesis‐related genes, indicating transcriptional repression of these genes. The observed nuclear remodeling, characterized by Lamin A/C disorganization, directly couples TRPV2‐mediated calcium overload to chromatin compaction, establishing a feedforward loop that perpetuates osteogenic suppression. Crucially, pharmacological TRPV2 inhibition not only normalized calcium flux but also reversed heterochromatin repositioning at osteogenic loci. These findings position TRPV2 as a biophysical rheostat that transduces extracellular viscosity into nuclear plasticity, ultimately impeding osteogenesis in the diabetic marrow microenvironment [49, 50]. The conserved nature of this mechanism across mechanopathologies suggests broad therapeutic potential for TRPV2 modulation in disorders of mechano‐epigenetic dysregulation.

Our findings unveil a hierarchical mechanoepigenetic axis wherein diabetic hyperviscosity activates TRPV2‐mediated calcium signaling, driving cytoskeletal‐nuclear uncoupling and chromatin compaction. This pathway not only elucidates the biomechanical basis of diabetic osteopathy but also provides a therapeutic rationale for targeting TRPV2 or modulating nuclear mechanoadaptation. It should be noted, however, that our current in vitro model primarily recapitulates the hyperviscous aspect of the diabetic marrow niche. Key pathophysiological features, such as AGEs accumulation and the hypoxic/acidic microenvironment, were not incorporated in this study. These factors may synergistically influence TRPV2 activity and BMSC function. Furthermore, the specific contribution of nuclear‐cytoskeletal linker proteins, such as those within the LINC complex, to the observed mechanical uncoupling remains to be fully elucidated. Future studies employing advanced 3D biomaterial‐based culture systems that concurrently mimic viscosity, biochemical abnormalities, and spatial constraints will be essential to fully capture the pathophysiology of diabetic osteopathy. In this context, future interventions may explore pharmacological TRPV2 inhibition, viscosity‐reducing agents, or chromatin‐modifying strategies to restore BMSCs regenerative capacity. By bridging extracellular fluid dynamics to nuclear architecture, this work advances our understanding of how diabetic microenvironments subvert stem cell plasticity through integrated biophysical and epigenetic mechanisms.

## 4 Experimental Section

### 4.1 Animal Models and Experiment

Male Sprague‐Dawley rats (SPF grade) were stratified into two cohorts: In vivo studies utilized animals weighing 180–200 g (6 weeks old), while in vitro experiments employed 90–120 g rats (4 weeks old), all procured from the Experimental Animal Center of Chongqing Medical University (License: SYXK(Yu)2019‐0001). Following a 7‐day acclimatization period with ad libitum access to water and standard chow, diabetic induction was initiated in accordance with institutional ethical guidelines (Approval No. CQHS‐REC‐2024‐178, Ethics Committee of CQMU Stomatological Hospital).

### 4.2 Diabetic Model Establishment

Hyperglycemia was triggered by intraperitoneally administering streptozotocin (STZ, 60 mg kg^−1^ dissolved in 100 mM citrate buffer). A fasting blood glucose concentration above 16.67 mM at 72 h after administration indicated successful induction of diabetes. Non‐diabetic controls consisted of healthy rats matched by age.

### 4.3 Calvarial Bone Defect Experiment

Healthy SD rats were designated as the Control group, while those exhibiting glucose fluctuations constituted the diabetes mellitus (DM) group. Under sterile conditions and general anesthesia, bilateral calvarial defects (4.5 mm in diameter) were precisely created in the parietal bones using a trephine drill. A single prophylactic dose of penicillin was given post‐surgery to prevent infection. To assess therapeutic effects, two additional groups were included: 1) The Prob group (WT controls) received intraperitoneal Prob (5 mg kg^−1^) every 12 h for 7 days, starting immediately after surgery; 2) The SKF group (DM rats) was treated with subcutaneous SKF (10 mg kg^−1^) once daily for 4 weeks, beginning at the time of defect induction.

### 4.4 Micro‐CT Imaging and Analysis

At designated time points (4 and 8 weeks post‐operation), animals were euthanized using CO_2_ inhalation followed by cervical dislocation. Calvarial samples were fixed in 4% paraformaldehyde for 24 h, then using a high‐resolution micro‐CT system (SCANCO, Switzerland) with a voxel size of 17.5 µm, 70 kV voltage, 800 µA current to scan samples. The images were reconstructed into 3D structures via N‐Recon software for morphometric assessment. Evaluated bone morphology metrics included bone volume to total volume ratio (BV/TV), trabecular number (Tb.N), and trabecular separation (Tb.Sp) at the defect site.

### 4.5 Cell Culture

Primary rBMSCs were isolated from age‐matched healthy and DM rats following established protocols, with minor modifications. Briefly, bone marrow was aseptically flushed from dissected femurs and tibiae using α‐MEM medium (Gibco, USA) supplemented with 10% heat‐inactivated fetal bovine serum (FBS; MedChemExpress, USA) and 1% penicillin‐streptomycin (HyClone, USA). The medium was refreshed every 48 h, and cells were subcultured at 80%–90% confluence using 0.25% trypsin‐EDTA (1:3 split ratio). For viscosity modulation experiments, we selected methylcellulose, a proven biocompatible material to mimic high viscosity environment [20], and diluted a 3% stock solution of methylcellulose (R&D Systems, USA) stepwise with complete α‐MEM to achieve final concentrations ranging from 0% to 2% (w/v). Pharmacological treatments included: Cytochalasin D (MedChemExpress, HY‐N6682), Probenecid (MedChemExpress, HY‐B0545) and SKF‐96365 hydrochloride (MedChemExpress, HY‐100001). Treatments were administered 24 h prior to functional assays unless otherwise specified.

### 4.6 Cell Viability

Cellular activity was quantified using the CCK‐8 assay (Sigma–Aldrich, USA). Briefly, rBMSCs (5 × 10^3^ cells well^−1^) were seeded in 96‐well plates and exposed to methylcellulose gradients (0%–2%) for 24 h. At designated intervals, 10% (v/v) CCK‐8 reagent pre‐equilibrated to 37 °C was introduced to each well, followed by 4 h incubation under standard culture conditions. Reaction supernatants were transferred to fresh plates to avoid optical interference from methylcellulose, and absorbance was measured at 450 nm (reference wavelength: 630 nm) using a NanoQuant Infinite M200 Pro microplate reader (Tecan, Switzerland). Post‐assay, cells were replenished with fresh medium for longitudinal monitoring at 48 h. Three independent biological replicates were performed with sextuplicate technical repeats.

### 4.7 Atomic Force Microscopy

Bone marrow samples were fixed by immersion in 4% paraformaldehyde and subsequently embedded in optimal cutting temperature (OCT) medium (Tissue‐Tek, USA) for cryosectioning. Following rapid freezing in liquid nitrogen‐cooled isopentane, blocks were sectioned into 40‐µm‐thick slices using a cryostat (NX50, Thermo Fisher Scientific, USA) maintained at −20 °C chamber temperature. For each experimental group, four biological replicates were analyzed with duplicate technical sections per specimen. rBMSCs were plated on collagen‐coated coverslips and cultured under standard conditions for 72 h to achieve optimal adhesion. Prior to atomic force microscopy (AFM) measurement, coverslips were transferred to HEPES‐buffered physiological solution maintained at 37 °C.

### 4.8 Rheometry Analysis

Rheological properties of bone marrow and cell culture medium samples were analyzed using a strain‐controlled rotational rheometer equipped with a Peltier temperature control unit. For each measurement, the upper plate was first brought into contact with the sample, applying a nominal initial force of 0.01 N (∼ 300 Pa) to ensure proper adhesion between the sample and the plates. Measurements were performed under a steady shear rate (30–200 rad s^−1^) over a duration of 600 s.

### 4.9 In Vitro Osteoblastic Differentiation

rBMSCs were plated at a density of 2 × 10⁴ cells per well in 24‐well plates and cultured in osteoinductive medium (α‐MEM supplemented with 10% FBS, 10 nM dexamethasone, 50 µg mL^−1^ L‐ascorbic acid, and 10 mM β‐glycerophosphate). After 7 days of induction, total RNA was isolated to assess the expression of genes related to osteogenic differentiation.

### 4.10 ALP and ARS Staining

On day 7 of culture under osteogenic conditions, BMSCs were subjected to ALP activity assessment using BCIP/NBT Chromogenic Substrate Kit (Roche Diagnostics, Switzerland). Following 28 days of osteogenic induction, calcium deposition in the ECM was quantified through ARS staining (Sigma–Aldrich, USA). All cellular morphological observations were documented using an EVOS Imaging System (Thermo Fisher Scientific, USA) under standardized illumination conditions.

### 4.11 Cell Migration Assay

For the scratch‐based planar migration assay, BMSCs were seeded onto experimental substrates in 12‐well culture plates and allowed to form confluent monolayers. Linear wounds were created using a sterile p200 pipette tip, followed by immediate replacement with serum‐free medium to eliminate proliferation interference. Migration was monitored at 2 h intervals over a 24 h period using phase‐contrast microscopy. Quantitative analysis was performed with ImageJ software. Cell migration was also evaluated using Transwell inserts equipped with 3 µm pore polycarbonate membranes (Corning Costar, USA). After 24 h of incubation under defined conditions, cells that had migrated and attached to the underside of the membrane were fixed and stained with 0.5% crystal violet (CV; MilliporeSigma, USA) for 30 min at room temperature. Excess stain was removed by washing three times with PBS before imaging using an EVOS Imaging System (Thermo Fisher Scientific).

### 4.12 SA‐β‐Gal Staining

Cellular senescence was assessed using a Senescence β‐Galactosidase Staining Kit (Beyotime, C0602) to visualize SA‐β‐Gal activity. Images were acquired using the EVOS imaging system.

### 4.13 Cell Volume Measurement

BMSCs were cultured in µ‐Slide 24‐well glass‐bottom plates pre‐coated with experimental substrates. After 24 h, the cells were stained with 5 nM DiO (3,3′‐dioctadecyloxacarbocyanine perchlorate) dye for 20 min to label the cell membranes. Subsequently, the live‐cell nuclear dye Hoechst was added for 3 min to stain the nuclei. Cells were subsequently fixed in 4% paraformaldehyde for 10 min. Samples were then visualized and imaged using a confocal microscope.

### 4.14 Morphological Analysis

BMSCs were cultured for 48 h and fluorescence images were taken, cell images were segmented using ImageJ software to identify cell boundaries, and to measure four shape‐dependent cellular morphological features: cell area, cell circularity, cell aspect ratio, cell solidity. Using a dimensionality reduction algorithm called UMAP space to compress above features into 2D space. Classify cells into five different morphological subtypes with unsupervised k‐Means clustering.

### 4.15 Immunofluorescence Staining

BMSCs were fixed with 4% paraformaldehyde for 10 min, followed by permeabilization using Triton X‐100 (Beyotime, P0096). After blocking with 5% goat serum (Zhongshan, China) for 1 h, cells were incubated overnight at 4 °C with the designated primary antibodies: Lamin A/C (Santa Cruz, sc‐376248, 1:300), YAP (Cell Signaling Technology, 14074S, 1:500), TRPV2 (Alomone Labs, ACC‐032, 1:200), vinculin (Abcam, ab129002, 1:200), CPLA2 (Bioss, bs‐20212R, 1:100), Histone H3 (tri methyl K9) (PTM BIO, PTM‐616, 1:200), and Histone H3 (acetyl K14) (PTM BIO, PTM‐113RM, 1:200). After three washes, cells were treated with Alexa Fluor 488/594‐labeled secondary antibodies for 1 h, then counterstained with DAPI (Beyotime, C1002) for 5 min to visualize nuclei. Fluorescence images were acquired using a confocal microscope, and signal intensities were analyzed with ImageJ software.

### 4.16 3D Confocal Imaging

BMSCs were seeded into a fibronectin‐coated Lab‐Tek II 8‐well chamber coverglass (Thermo Fisher Scientific, US) at a density of ∼ 5000 cells well^−1^ and incubated at 37 °C for 3 days prior to the experiment. BMSCs were fixed in 4% paraformaldehyde for 10 min, then permeabilized with Triton X‐100 (Beyotime, P0096). F‐actin was labeled using Actin‐Tracker Red‐Rhodamine (Beyotime, C2007S) for 1 h, followed by nuclear counterstaining with DAPI (Beyotime, C1002) for 5 min. Fluorescence images were acquired using a confocal microscope. The images were scanned at a 1024 × 1024 (pixel × pixel) resolution and 1 µm z‐step size.

### 4.17 F‐Actin Staining

BMSCs were fixed in 4% paraformaldehyde for 10 min, then permeabilized with Triton X‐100 (Beyotime, P0096). F‐actin was labeled using Actin‐Tracker Red‐Rhodamine (Beyotime, C2007S) for 1 h, followed by nuclear counterstaining with DAPI (Beyotime, C1002) for 5 min. Fluorescent images were captured using a confocal microscope.

### 4.18 Protein Extraction and Western Blotting

Cells were lysed in RIPA buffer containing protease and phosphatase inhibitors. After centrifugation (12000×g, 15 min, 4 °C), protein levels in the supernatant were quantified using a BCA kit (Thermo Fisher Scientific) with BSA as standard. Proteins (10–50 µg lane^−1^) were separated by SDS‐PAGE and transferred to PVDF membranes (Millipore). Membranes were blocked with 5% BSA in TBST for 1 h, incubated with primary antibodies overnight at 4 °C, and then with secondary antibodies (1:5000; CST) for 2 h. Signals were detected using an ECL system (Bio‐Rad). Primary antibodies: F‐actin (Abcam, ab205), G‐actin (Abcam, ab200046), p‐CaMKII (Abcam, ab124880), p‐cofilin (Abcam, ab283500), cofilin (Abcam, ab54532), TRPV2 (Alomone Labs, ACC‐032), Histone H3 (tri methyl K9) (PTM BIO, PTM‐616), and Histone H3 (PTM BIO, PTM‐6621), Histone H3 (tri methyl K27) (PTM BIO, PTM‐647RM), Histone H3 (tri methyl K4) (PTM BIO, PTM‐5019), GAPDH (Abcam, ab181602).

### 4.19 Calcium Measurements

BMSCs were incubated at 37 °C for 30 min with 2 µM Fluo‐4 AM (Beyotime, S1060) in Ca^2^⁺/Mg^2^⁺‐free HBSS‐HEPES buffer, followed by three washes with the same buffer. Ca^2^⁺ oscillations were recorded via time‐lapse imaging every 2 s for 10 min. Image analysis was conducted using LAS X software.

### 4.20 Flow Cytometry

BMSCs were loaded with 2 µM Fluo‐4 AM (Beyotime, S1060) in Ca^2^⁺/Mg^2^⁺‐free HBSS‐HEPES buffer at 37 °C for 30 min. After incubation, cells were washed three times with the same buffer. Calcium oscillations were recorded using time‐lapse imaging, with images captured every 2 s over a 10 min period, as previously described. Image analysis was conducted using FlowJo software (v10.8.1).

### 4.21 Quantitative Real‐Time PCR (qRT‐PCR)

Total RNA was extracted using Trizol reagent (Thermo Fisher Scientific). qRT‐PCR was performed on a ProFlex PCR system (Thermo Fisher) with TB Green PCR Master Mix (Takara). GAPDH was used as the internal control. Primer sequences for target genes were listed in Table S1.

### 4.22 Knockdown of Trpv2 Expression by siRNA

For gene silencing, BMSCs from diabetic or MHC groups were transfected with si‐Trpv2 or a negative control siRNA (siNC) using Lipofectamine RNAiMAX (Invitrogen, Thermo Fisher Scientific, USA). Cells were plated the evening before transfection, the following day, the siRNA–lipid complex was added. After 6 h, Opti‐MEM was replaced with fresh complete medium. Protein expression was assessed 48 h later. The sequences of the small interfering RNA were listed in Table S2.

### 4.23 Transmission Electron Microscopy

After treatment, 1 × 10⁶ cells were fixed in 4% glutaraldehyde (Solarbio) for 24 h at 4 °C, then incubated with 1% osmium tetroxide (Electron Microscopy Sciences) and dehydrated through an ethanol series. The samples were embedded in Araldite resin, and 85 nm thin sections were stained with uranyl acetate and lead citrate. Ultrastructural analysis was performed using transmission electron microscopy (TEM) (FEI, Hillsboro, Oregon) at 80 kV.

### 4.24 ChIP‐Seq

Cells were cross‐linked with 1% formaldehyde and incubated for 10 min, followed by the addition of glycine to quench the cross‐linking. Cells were washed with PBS, scraped, and collected by centrifugation. The cell pellets were resuspended in pre‐chilled ChIP lysis buffer (50 mM HEPES, 150 mM NaCl, 2 mM EDTA, 0.1% Na‐deoxycholate, 0.5% Triton X‐100) with protease and/or phosphatase inhibitors, and sonicated (20 min, 30 seconds on, 30 seconds off). A 20 µL aliquot of input DNA was saved. After sonication, debris was removed by centrifugation, and the supernatant was collected. For immunoprecipitation, 2–5 µg of antibody was added to the sample and incubated overnight. The antibody‐bound complexes were incubated with pre‐blocked magnetic A/G beads for 2–4 h at 4 °C. The beads were washed with high‐salt and low‐salt buffers, followed by elution with ChIP elution buffer. DNA was purified after treatment with proteinase K, and the concentration was measured using Qubit. The library was then constructed. The antibodies used in the ChIP assay were H3K9me3 (#13969T, CST, 4 µg test^−1^).

### 4.25 Abaqus Simulation

The influence of medium viscosity on cell confining stress during stress relaxation was analyzed using the finite element tool (ABAQUS 6.14‐1). An ellipsoidal‐shaped cell and a cuboid culture medium were constructed as a 3D deformable solid model. The cell radius was derived from experimentally measured volume data. Positioned at the center of the medium, the model employed symmetric boundary conditions to minimize computational load. The cell was modeled as an elastic material with a modulus of 500 Pa and Poisson's ratio of 0.49, while the medium was treated as viscoelastic, with the relaxation modulus fitted to a Prony series and the same Poisson's ratio. G(t): Relaxation modulus (Prony series: G(t) = G∞+∑Giexp(‐t/τi); where Gi: shear modulus, τi: relaxation time.

### 4.26 Statistical Analysis

Comparisons between two groups were made using a two‐tailed Student's t‐test. For multiple groups, one‐way ANOVA followed by Tukey's post hoc test was used. Results are presented as mean ± standard deviation (S.D.), with *p < 0.05, **p < 0.01, and ***p < 0.001 indicating significance levels, and ns indicating no significant difference. All analyses were conducted using Origin (2021) and GraphPad Prism (10.1.1), with at least three independent replicates.

## Conflicts of Interest

The authors declare no conflict of interest.

## Supporting information




**Supporting File**: advs73471‐sup‐0001‐SuppMat.pdf.

## Data Availability

The data that support the findings of this study are available from the corresponding author upon reasonable request.
