# Supplementary material for: Hyperviscous Diabetic Bone Marrow Niche Impairs BMSCs Osteogenesis via TRPV2‐Mediated Cytoskeletal‐Nuclear Mechanotransduction
Source: Adv Sci (Weinh). 2025 Dec 22;13(13):e09056. doi: 10.1002/advs.202509056 (PMC12955904; doi:10.1002/advs.202509056)
Supplement: Supplementary file 1 — Supporting File: advs73471‐sup‐0001‐SuppMat.pdf. [file ADVS-13-e09056-s001.docx]

Supporting Information

**Hyperviscous Diabetic Bone Marrow Niche Impairs BMSCs Osteogenesis via TRPV2-Mediated Cytoskeletal-Nuclear Mechanotransduction**

Yao Wen, Xinhui Zheng, Jieliu Li, Minyu He, Dongqi Fan, Xingyu Zhu, Qiming Zhai*, Liangjing Xin*, Tao Chen*

**Figure S1**
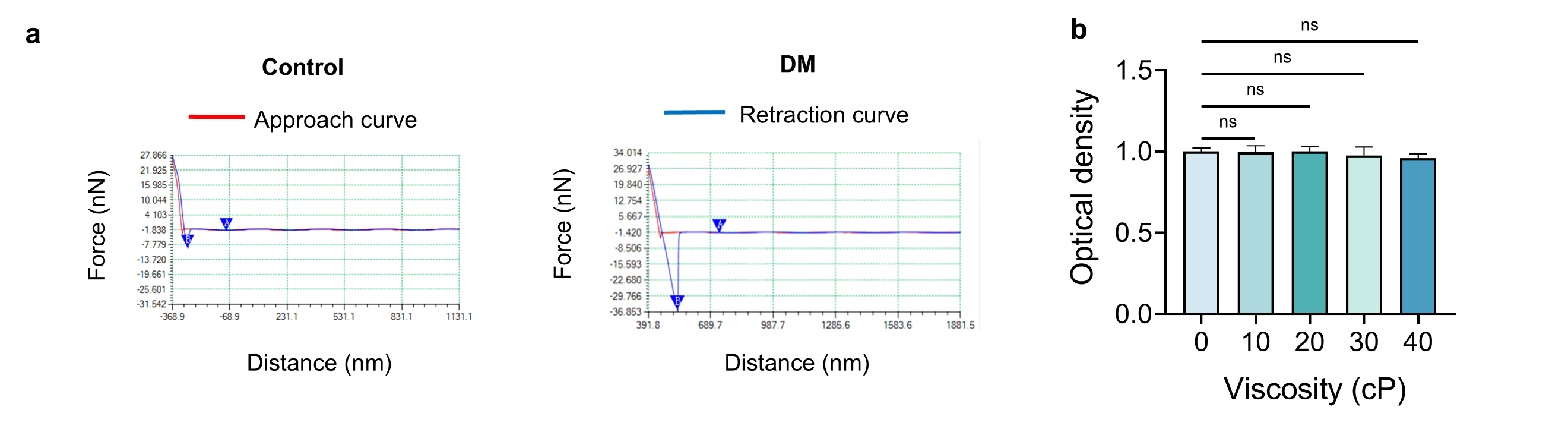


**Figure S1.** a) AFM Retraction Curves for bone marrow viscosity (n = 3). b) CCK-8 proliferation assay (n = 3). All data are represented as mean ± standard deviation; *p < 0.05, **p < 0.01, ***p < 0.001. One-way ANOVA followed by Tukey’s post hoc test was used for comparisons in b).

**Figure S2**


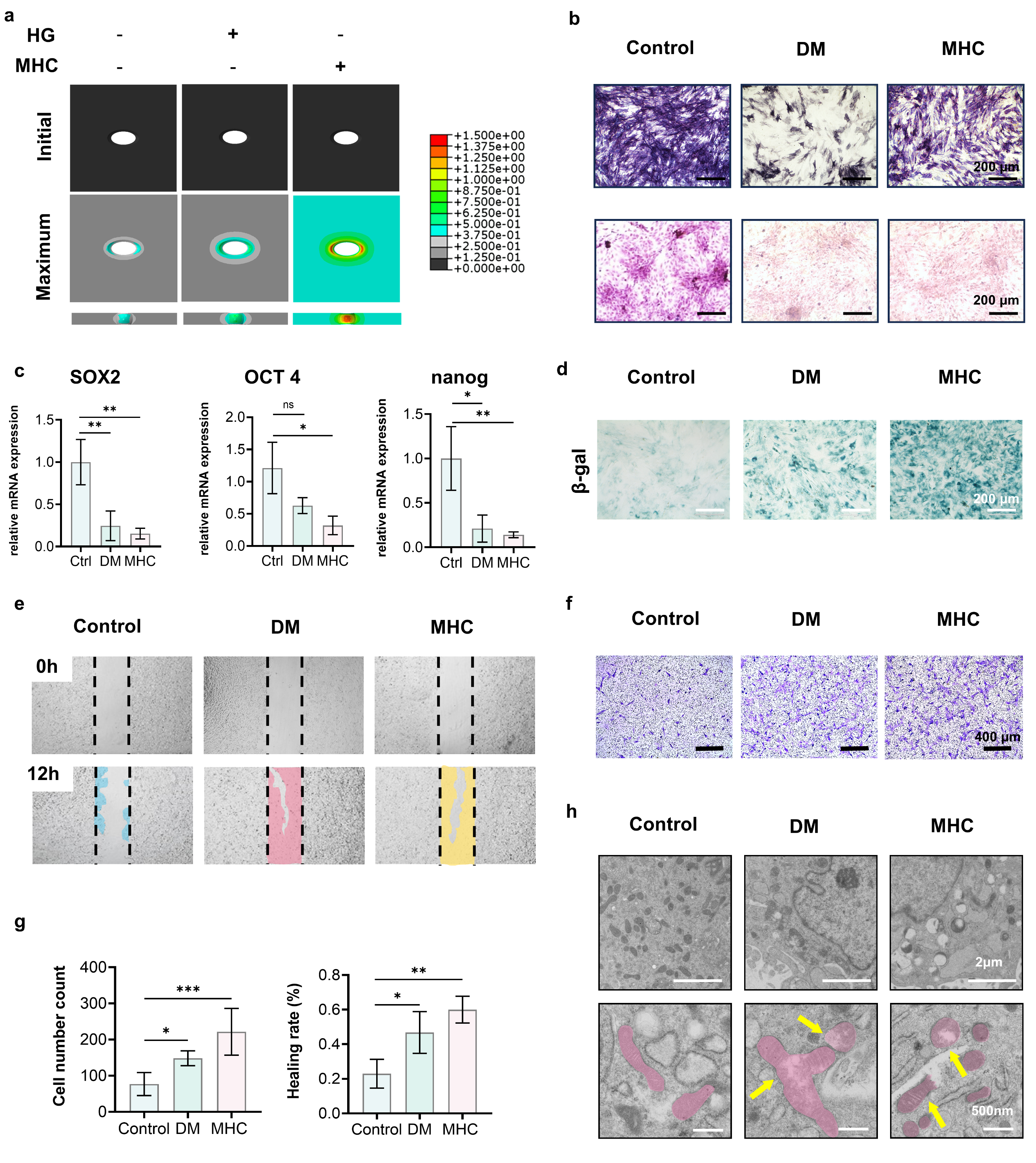


**Figure S2.** a) ABAQUS simulation to model confining stress during cell volumetric growth with different culture condition. b) ALP and ARS staining after osteogenic differentiation induction for 7 and 21 days. Scale bar: 200 μm (n = 3). c) Relative gene expressions of stemness genes (SOX2, OCT4, nanog) (n = 3). d) SA-β-gal staining of BMSCs. Scale bar: 200 μm (n = 3). e) Cell scratch assay (n = 3). f) Transwell migration. Scale bar: 400 μm (n = 3). g) Quantitative analysis of scratch and migration assays (n = 3). h) Representative TEM images of BMSCs. Structures colored pink indicates mitochondria. Scale bar: 2 μm, 500 nm (n = 3). All data are represented as mean ± standard deviation; *p < 0.05, **p < 0.01, ***p < 0.001. One-way ANOVA followed by Tukey’s post hoc test was used for comparisons in c), g).

**Figure S3**


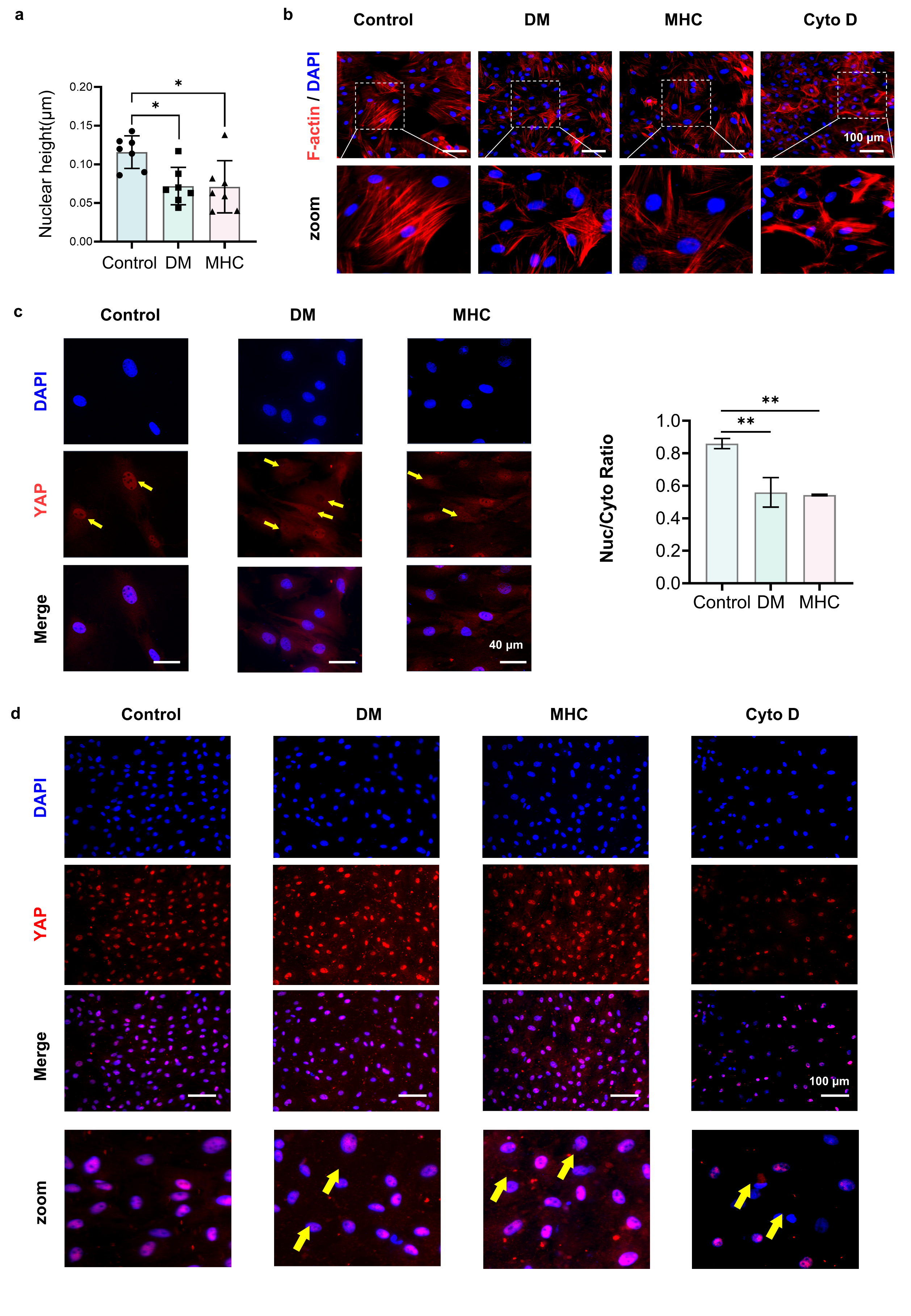


**Figure S3.** a) Statistical analysis of nuclear height (n = 7). b) Immunofluorescence image of F-actin and nuclear. Normalized fluorescence intensity profiles of F-actin across the nucleus (bottom). Scale bar: 100 μm (n = 3). c) Immunofluorescence staining of the YAP distribution. Scale bar: 40 μm (n = 3). d) Cyto D-treated cells show cytoplasmic YAP retention. Scale bar: 100 μm (n = 3). All data are represented as mean ± standard deviation; *p < 0.05, **p < 0.01. One-way ANOVA followed by Tukey’s post hoc test was used for comparisons in a), c).

**Figure S4**

**
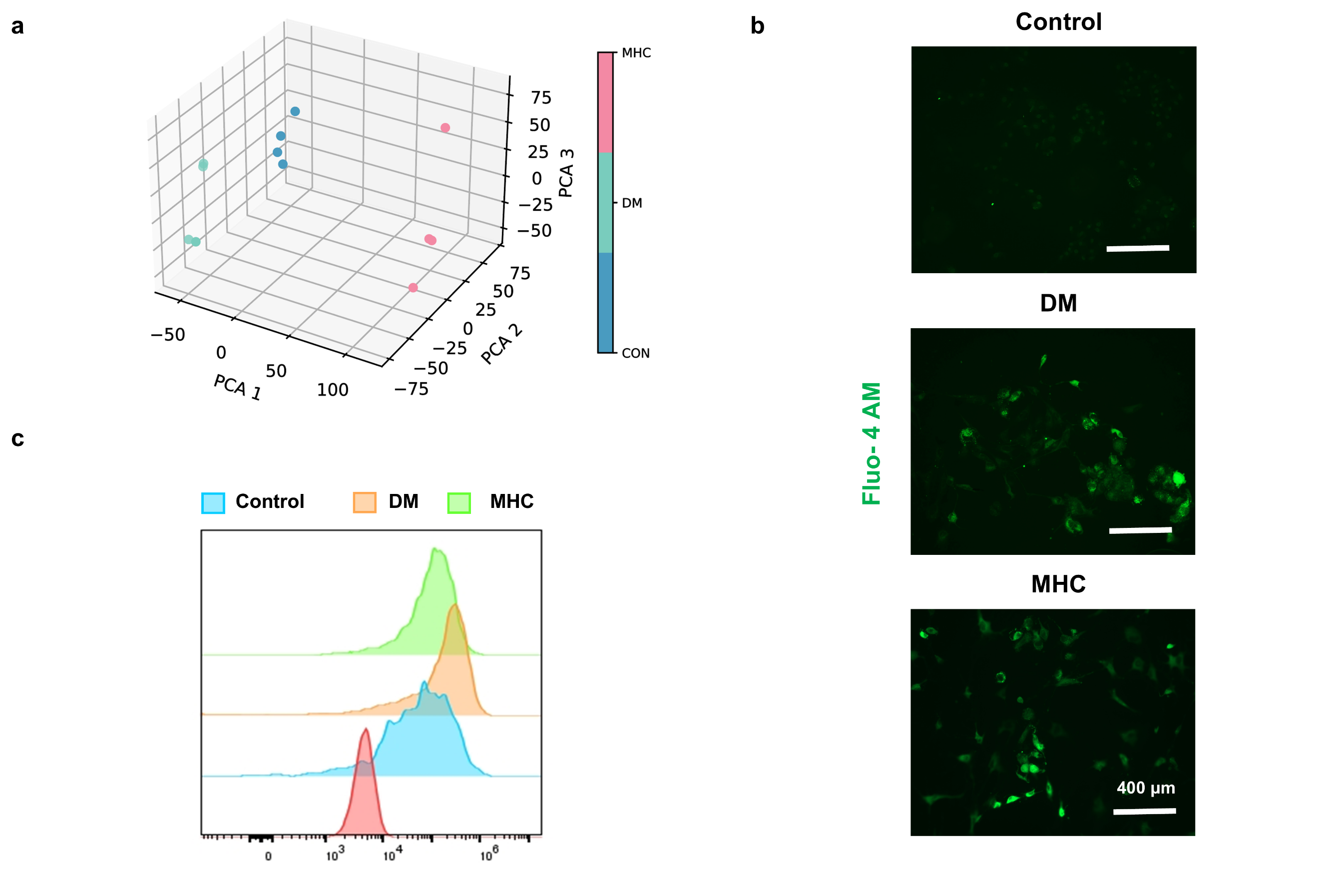
**

**Figure S4.** a) Principal component analysis (PCA) of RNA sequencing data (n = 4). b) Fluo-4 AM intensity profiles. Scale bar: 400 μm (n = 3). c) Flow cytometric quantification of intracellular Ca²⁺ levels (n = 3). Data are represented as mean ± standard deviation; *p < 0.05, **p < 0.01.

**Figure S5**


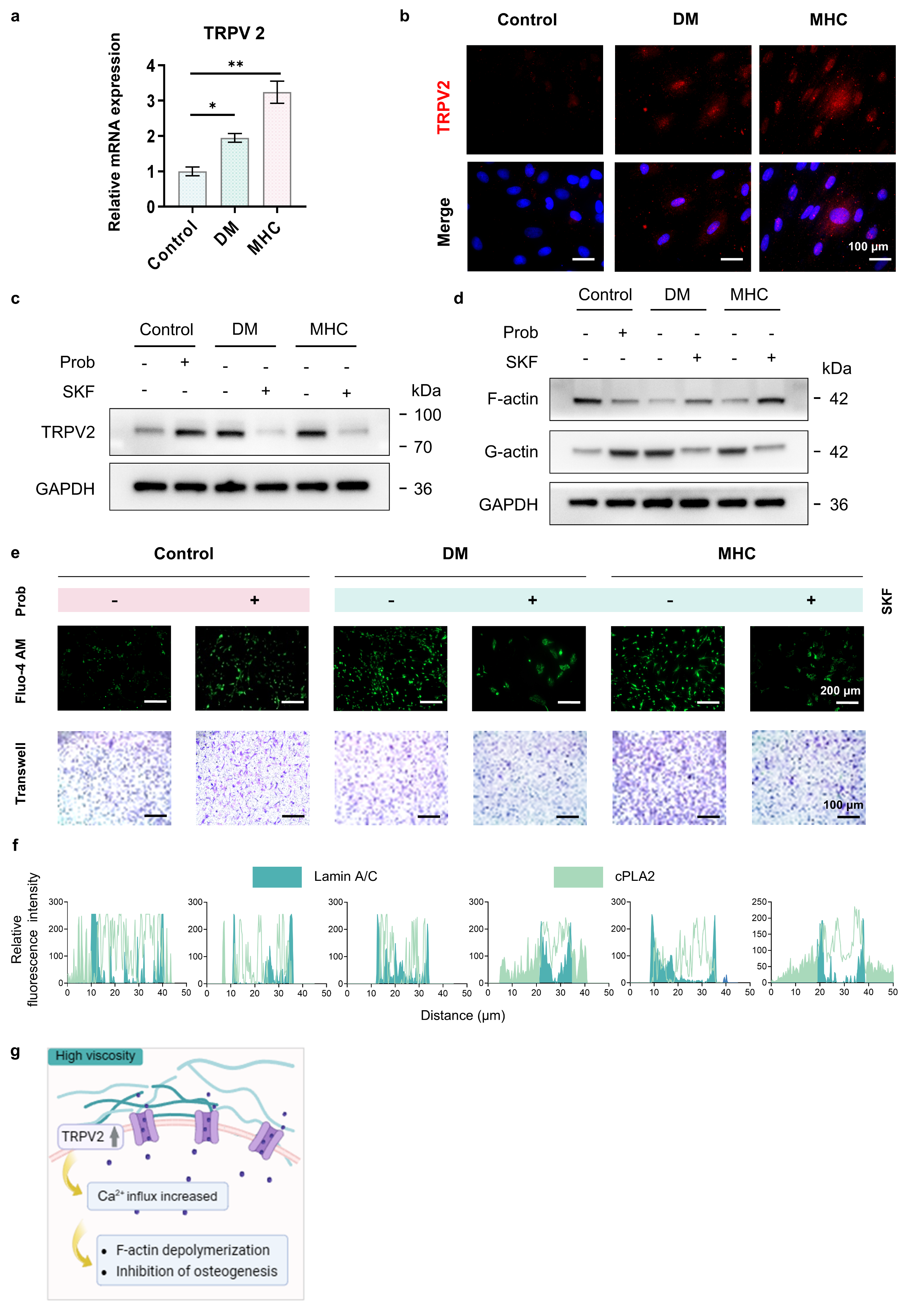


**Figure S5.** a) The relative mRNA expression level of TRPV2 (n = 3). b) Immunofluorescence image of TRPV2. Scale bar: 100 μm (n = 3). c) Expression of TRPV2 quantified by western blotting. d) Expression of F-actin and G-actin quantified by western blotting. e) Representative images of cytosolic calcium and transwell assay. Scale bar: 200, 100 μm (n = 3). f) Radial intensity heatmaps of cPLA2 and Lamin A/C. g) Proposed pathway under low and high viscosity condition. Data are represented as mean ± standard deviation; *p < 0.05, **p < 0.01. One-way ANOVA followed by Tukey’s post hoc test was used for comparisons in a).

**Figure S6**


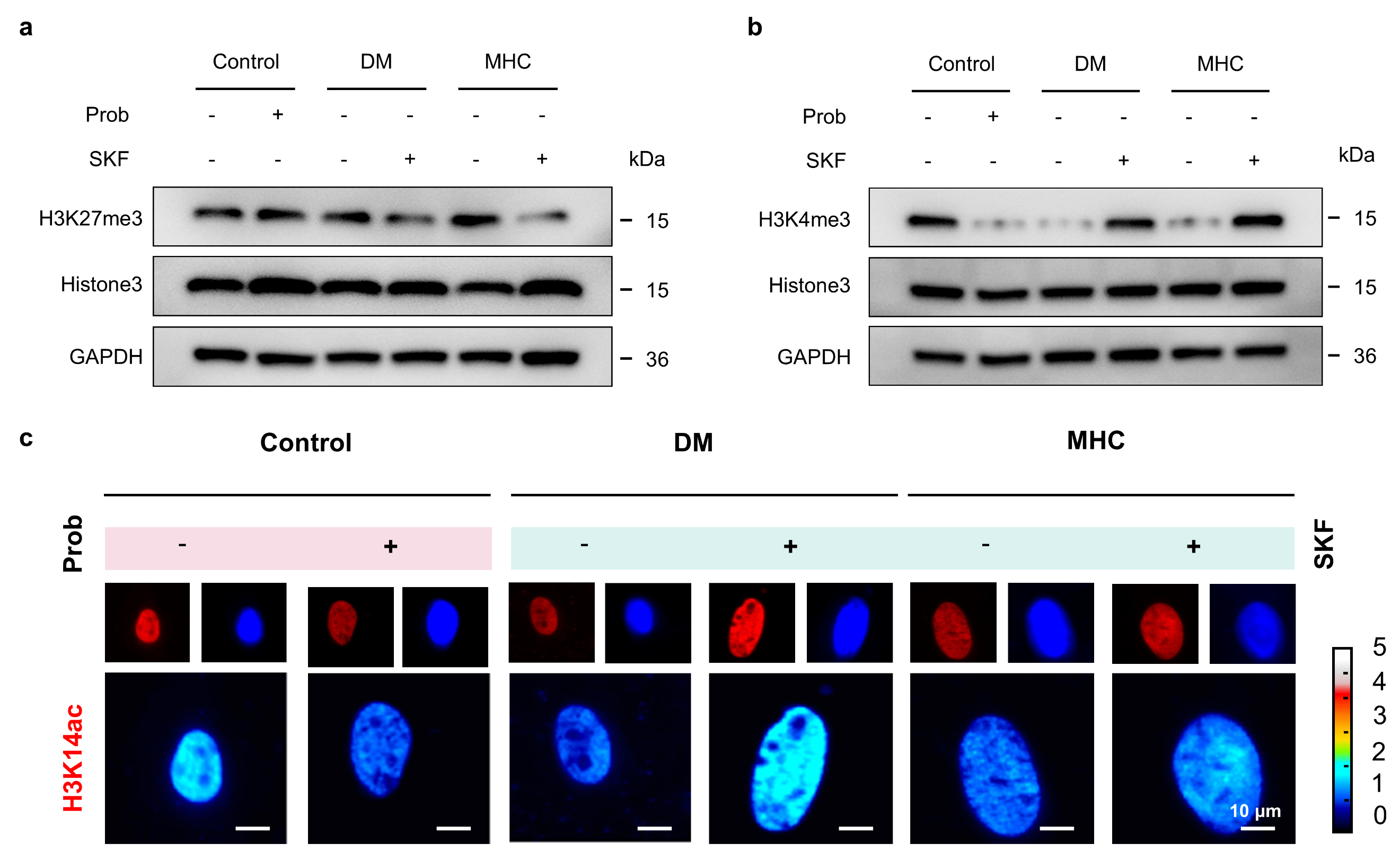


**Figure S6.** a-b) The expression of H3K27me3 and H3K4me3 proteins measured by western blotting. c) Representative immunofluorescence images of BMSCs stained for H3K14ac and counterstained with DAPI to visualize nuclei. Scale bar: 10 μm (n = 3).

**Figure S7**


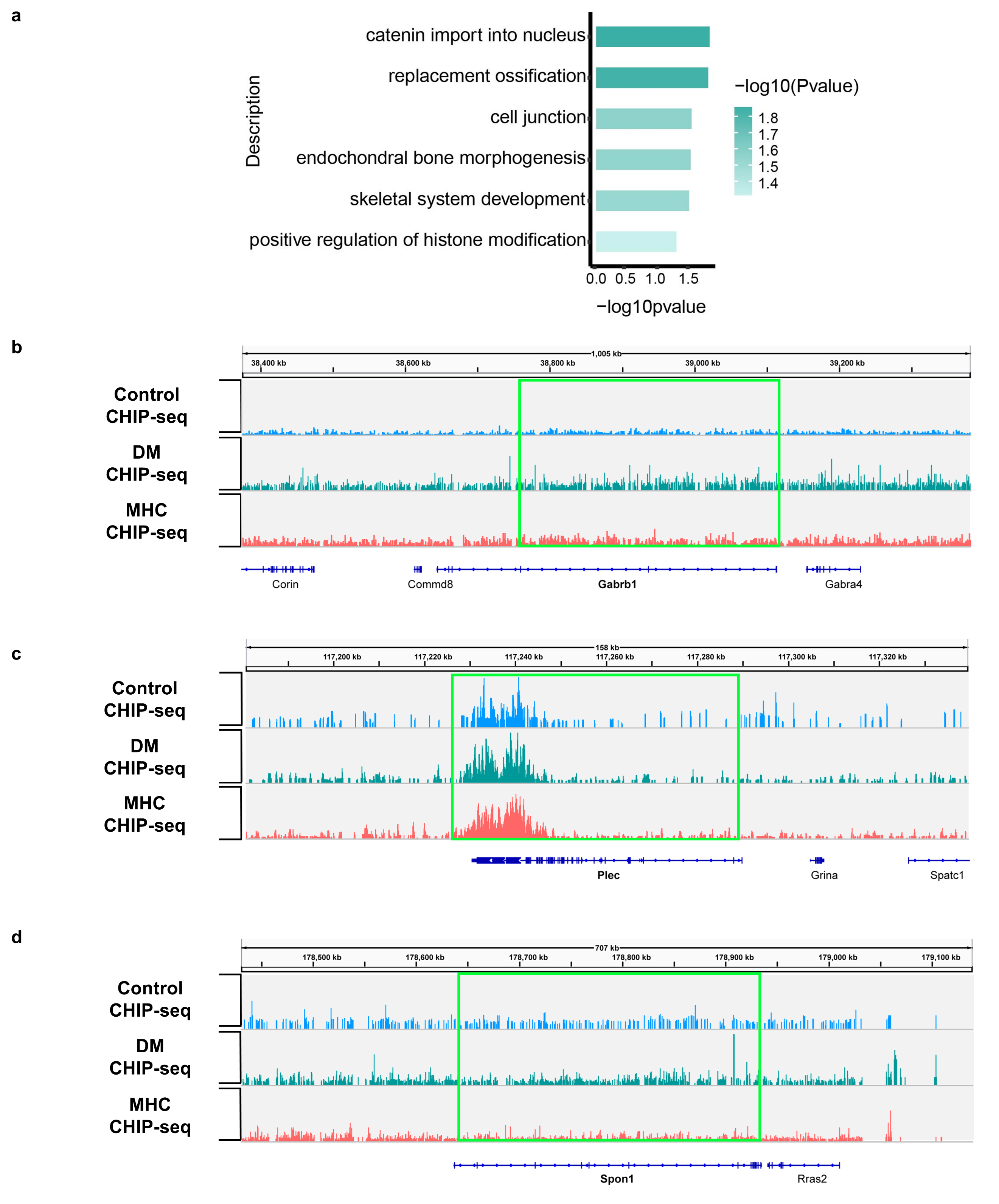


**Figure S7.** a) GO enrichment of DEGs in the DM group (n = 2). b-d) Representative IGV tracks demonstrating increased H3K9me3 binding enrichment within heterochromatic regions of the Gabrb1, Plec and Spon1 gene (n = 2).

**Figure S8**


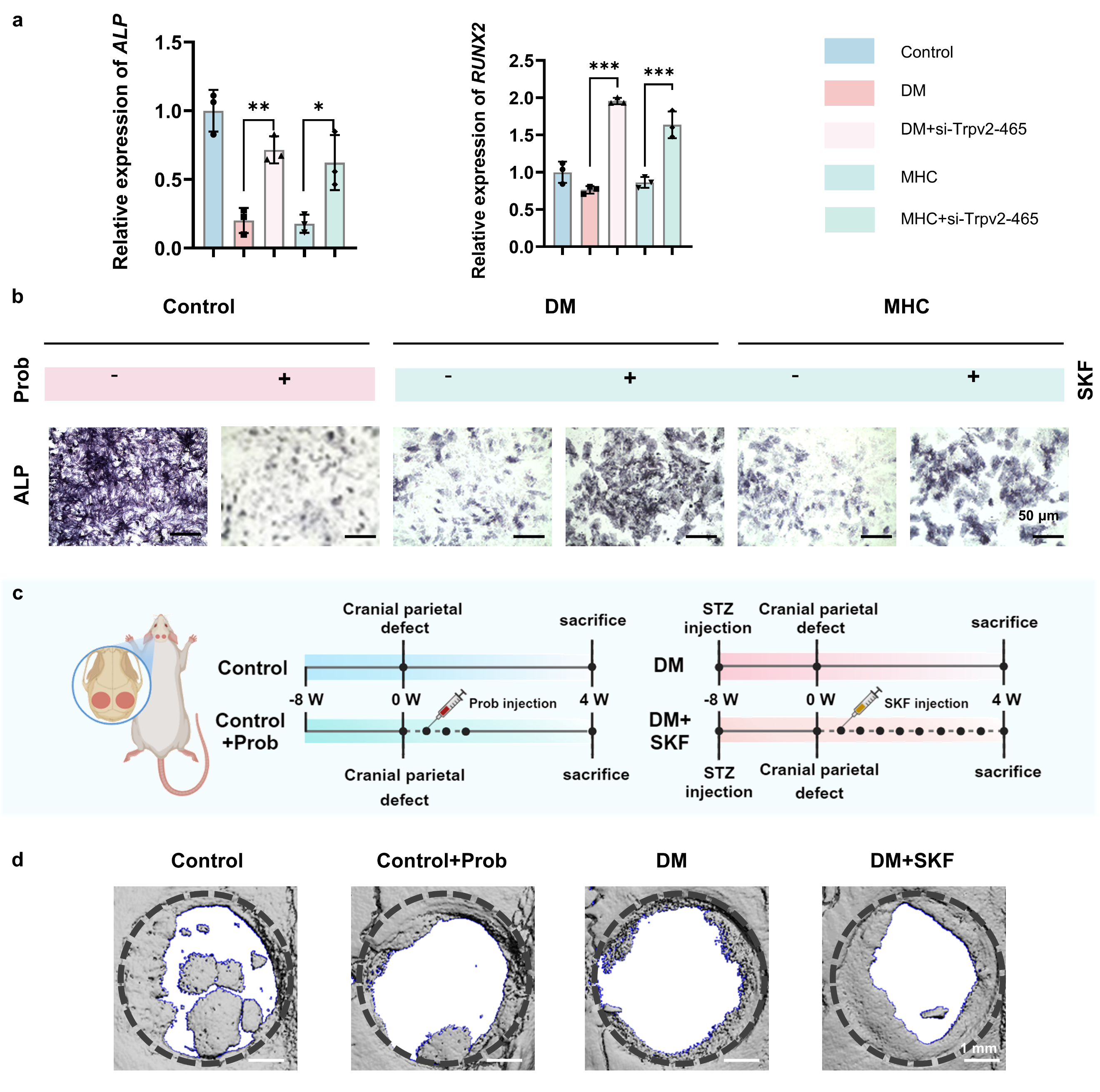


**Figure S8.** a) The relative mRNA expression levels of ALP and RUNX2 (n = 3). b) Representative images of ALP staining. Scale bar: 50 μm (n = 3). c) Schematic diagram of animal modeling. d) Micro-CT image of rat cranial parietal 4 weeks after cranial parietal modeling for cranial parietal bone sampling. Scale bar: 1 mm (n = 3). Data are represented as mean ± standard deviation; *p < 0.05, **p < 0.01, ***p < 0.001. One-way ANOVA followed by Tukey’s post hoc test was used for comparisons in a).

**Table S1.** Primers used for qRT-PCR.

| Gene | Sequence |
| --- | --- |
| GAPDH | F: GGCACAGTCAAGGCTGAGAATG  R: ATGGTGGTGAAGACGCCAGTA |
| Nanog | F: ACGCTGCTCCGCTCCATAAC R: CAAATTCGCCTCCAAATCACTG |
| Oct4 | F: GTGTTCAGCCAGACAACCATC R: CACCAGGGTCTCCGATTTG |
| Sox2 | F: GCCGAGTGGAAACTTTTGTC  R: CCGGGAAGCGTGTACTTATC |
| TRPV 2 | F: AGGAGCTGACTGGACTGCTA  R: GAGCCTTCTGTGTATGCCGA |
| ALP | F: CAGGTGTCGGAAGATGGGAAA  R: TACTGACGGAAGAAGGGGTGC |
| RUNX-2 | F: CATGGCCGGGAATGATGAG  R: CATGGCCGGGAATGATGAG |

**Table S2.** The sequence of small interfere RNA

| siRNA | Sense（5’-3’） | Antisense (5’-3’) |
| --- | --- | --- |
| si-*Trpv2*-144 | AGAUCAAAGUGAACCUCAATT | UUGAGGUUCACUUUGAUCUTT |
| si-T*rpv2*-465 | AGUGCAUCGAUGAGUUCUATT | UAGAACUCAUCGAUGCACUTT |
| si-*Trpv2*-415 | GCUGCAGAUUGACAAGGAUTT | AUCCUUGUCAAUCUGCAGCTT |
